# Benchmarking health system performance across states in Nigeria: a systematic analysis of levels and trends in key maternal and child health interventions and outcomes, 2000–2013

**DOI:** 10.1186/s12916-015-0438-9

**Published:** 2015-09-02

**Authors:** Alexandra Wollum, Roy Burstein, Nancy Fullman, Laura Dwyer-Lindgren, Emmanuela Gakidou

**Affiliations:** Institute for Health Metrics and Evaluation, University of Washington, 2301 5th Ave, Suite 600, Seattle, WA 98121 USA

**Keywords:** Coverage, Inequalities, Maternal and child health, Nigeria, Subnational benchmarking, Under-5 mortality

## Abstract

**Background:**

Nigeria has made notable gains in improving childhood survival but the country still accounts for a large portion of the world’s overall disease burden, particularly among women and children. To date, no systematic analyses have comprehensively assessed trends for health outcomes and interventions across states in Nigeria.

**Methods:**

We extracted data from 19 surveys to generate estimates for 20 key maternal and child health (MCH) interventions and outcomes for 36 states and the Federal Capital Territory from 2000 to 2013. Source-specific estimates were generated for each indicator, after which a two-step statistical model was applied using a mixed-effects model followed by Gaussian process regression to produce state-level trends. National estimates were calculated by population-weighting state values.

**Results:**

Under-5 mortality decreased in all states from 2000 to 2013, but a large gap remained across them. Malaria intervention coverage stayed low despite increases between 2009 and 2013, largely driven by rising rates of insecticide-treated net ownership. Overall, vaccination coverage improved, with notable increases in the coverage of three-dose oral polio vaccine. Nevertheless, immunization coverage remained low for most vaccines, including measles. Coverage of other MCH interventions, such as antenatal care and skilled birth attendance, generally stagnated and even declined in many states, and the range between the lowest- and highest-performing states remained wide in 2013. Countrywide, a measure of overall intervention coverage increased from 33% in 2000 to 47% in 2013 with considerable variation across states, ranging from 21% in Sokoto to 66% in Ekiti.

**Conclusions:**

We found that Nigeria made notable gains for a subset of MCH indicators between 2000 and 2013, but also experienced stalled progress and even declines for others. Despite progress for a subset of indicators, Nigeria’s absolute levels of intervention coverage remained quite low. As Nigeria rolls out its National Health Bill and seeks to strengthen its delivery of health services, continued monitoring of local health trends will help policymakers track successes and promptly address challenges as they arise. Subnational benchmarking ought to occur regularly in Nigeria and throughout sub-Saharan Africa to inform local decision-making and bolster health system performance.

**Electronic supplementary material:**

The online version of this article (doi:10.1186/s12916-015-0438-9) contains supplementary material, which is available to authorized users.

## Background

Over the last two decades, Nigeria has documented both progress and challenges in improving the health of its population [1]. Although under-5 mortality decreased by 38% between 1990 and 2013, 14% of child deaths in the world still occurred in Nigeria in 2013 [2]. Minimal gains have occurred for maternal mortality over the past 25 years, with Nigeria’s maternal mortality ratio consistently hovering around 500 deaths per 100,000 live births since 1990 [3]. In 2013, 30% of the world’s malaria cases and deaths occurred within Nigeria’s borders [4].

Nigeria and its development partners have made several efforts to address the country’s health needs. To date, investments to scale up polio immunization campaigns and malaria control have been particularly large [5, 6]; for instance, the Global Polio Eradication Initiative (GPEI) plans to spend nearly $1.5 billion on efforts in Nigeria between 2013 and 2018 [7]. Beyond disease-specific programs, Nigeria has also enacted policies to improve access to and quality of health care [8–16]. In 2014, the National Health Bill was passed, aiming to strengthen Nigeria’s primary health care systems, bolster monitoring and evaluation capacities, and move toward universal health coverage through improved financial protections [17].

Health policies and programs are typically implemented in a phased manner in Nigeria, largely due to the country’s large size and decentralized health system [10, 13, 18]. State governments oversee health funding and logistic support, whereas local government areas (LGAs) are the geographic units from which primary health services are provided [10, 19]. Historically, LGAs have been under-funded and operate with less capacity to implement health initiatives than originally planned [20]. In combination with the country’s large and diverse population, it often takes years before interventions have reached all states. For instance, over a third of states rolled-out the pentavalent vaccine two years after it was originally introduced in the country [21], while mass distribution campaigns for long-lasting insecticide-treated nets (LLINs) occurred state by state between 2008 and 2014 [6, 22, 23]. This highlights the need to track and assess trends in intervention coverage and health outcomes at the subnational level in Nigeria.

A number of other populous countries, such as Mexico and Brazil, have been successful in using subnational benchmarking exercises to inform policy decisions and program priorities [24–26]. Although efforts have been made to synthesize subnational health information in Nigeria, these data have not often been comparable over time, nor have they presented the country’s full geography [13, 27–32]. Recently, there have been efforts to develop a LGA-level tool for evaluating progress on health, education, and environmental indicators by Nigeria’s Millennium Development Goal (MDG) office [33], a critical step to improving subnational monitoring systems. However, this tool shows information for each indicator for only 1 year and thus cannot provide an understanding of trends over time – a vital component to capturing the effects of specific health policies and programs.

By synthesizing data from multiple sources, we provide the first-ever analysis of state-level trends for a range of Nigeria’s key maternal and child health (MCH) outcomes and interventions from 2000 to 2013.

## Methods

### Indicator selection

We identified 20 MCH outcomes and interventions based on their relevance to Nigeria’s health priorities and data availability at the state-level. These indicators included child health outcomes (all-cause under-5 mortality; the proportion of children under 5 who were underweight; the prevalence of wasting among children under 5; the prevalence of stunting among children under 5), malaria interventions (household ownership of at least one insecticide-treated net [ITN]; ITN use by children under 5; the proportion of households owning at least one ITN or having received indoor residual spraying [IRS]; intermittent preventative therapy for malaria during pregnancy, two doses [IPTp2]; the proportion of children who received artemisinin-based combination therapies [ACTs] in response to having a fever), immunizations (the Bacillus Calmette-Guérin [BCG] vaccine; measles vaccine; three doses of the diphtheria-pertussis-tetanus vaccine [DPT3]; three doses of the oral polio vaccine [OPV3]), and other key MCH interventions (one and four antenatal care visits [ANC1 and ANC4]; skilled birth attendance [SBA]; rates of in-facility deliveries [IFD]; exclusive breastfeeding [EBF]; modern contraception use; and the receipt of at least two doses of the tetanus toxoid vaccine during pregnancy). State-level data were not available for HIV/AIDS treatment. Table 1 provides definitions for each indicator. We focus on a subset of these indicators in this paper, but present results for all indicators in Additional files 1 and 2, as well as through an interactive data visualization tool [34].

**Table 1:** Definition of indicators

Indicator (abbreviation)	Definition	Data sources
Maternal and child health indicators		
Antenatal care (ANC1, ANC4)	The proportion of women aged 15–49 years who gave birth in the given year and had one/four or more antenatal visits attended by skilled personnel (doctor, nurse, midwife, or clinical officer) at a health facility during the corresponding pregnancy	DHS: 2003, 2008, 2013
		MICS: 2007, 2011
		LSS: 2003–2004
		CWIQ: 2006
Skilled birth attendance (SBA)	The proportion of women aged 15–49 years who gave birth in the given year and delivered with a skilled birth attendant (a doctor, nurse, midwife, or clinical officer)	DHS: 2003, 2008, 2013
		MICS: 2007, 2011
		CWIQ: 2006
		SMART: 2013
In-facility deliveries (IFD)	The proportion of women aged 15–49 years who gave birth in a given year and delivered at a health facility	DHS: 2003, 2008, 2013
		MICS: 2007, 2011
		CWIQ: 2006
Exclusive breastfeeding (EBF)	The proportion of children who were exclusively breastfed during their first six months after birth	DHS: 2003, 2008, 2013
		MICS: 1999, 2007, 2011
		GHS: 2006, 2007, 2010, 2012
Prevalence of modern contraceptive use	The proportion of women aged 15–49 years who reported using a modern method of contraception	DHS: 2003, 2008, 2013
		MICS: 1999, 2007, 2011
		GHS: 2006, 2007, 2010, 2012
		LSS: 2003–2004, 2009–2010
Percentage of women who received two or more doses of the tetanus toxoid vaccine during pregnancy	The proportion of women aged 15–49 years who received two or more doses of the tetanus toxoid vaccine during their last pregnancy	DHS: 2003, 2008, 2013
		MICS: 2007, 2011
Childhood immunizations		
Bacillus Calmette-Guérin immunization (BCG)	The proportion of children under 5 who were vaccinated against tuberculosis in the given year	DHS: 2003, 2008, 2013
		MICS: 1999, 2007, 2011
		LSS: 2003–2004, 2009–2010
Diphtheria-pertussis-tetanus immunization, three doses (DPT3)	The proportion of children aged 12–59 months old who received three doses of the diphtheria-pertussis-tetanus (DPT) vaccine in the given year	DHS: 2003, 2008, 2013
		MICS: 1999, 2007, 2011
		LSS: 2003–2004, 2009–2010
Measles immunization (Measles)	The proportion of children aged 12–59 months who received measles vaccination in the given year	DHS: 2003, 2008, 2013
		MICS: 1999, 2007, 2011
		LSS: 2003–2004, 2009–2010
Oral polio vaccine immunization, three doses (OPV3)	The proportion of children aged 12–59 months who received three doses of the oral polio vaccine in the given year	DHS: 2003, 2008, 2013
		MICS: 1999, 2007, 2011
		LSS: 2003–2004, 2009–2010
Malaria interventions		
Intermittent preventive therapy for malaria during pregnancy, two doses (IPTp2)	The proportion of women aged 15–49 years who gave birth in the given year and received at least two treatment doses of Fansidar (sulfadoxine/pyrimethamine) at antenatal care visits during the corresponding pregnancy	DHS: 2003, 2008, 2013
		MICS: 2007, 2011
		MIS: 2010
Indicator (abbreviation)	Definition	Data sources
Malaria interventions		
Household ownership of ITNs (ITN own)	The proportion of households that own at least one insecticide-treated net (ITN)	DHS: 2003, 2008, 2013
		MICS: 2011
		MIS: 2010
		LSS: 2009–2010
		Netmark: 2000, 2004, 2008
ITN use by children under 5 (ITN use)	The proportion of children under 5 who slept under an ITN the previous night	DHS: 2003, 2008, 2013
		MICS: 2007, 2011
		MIS: 2010
		LSS: 2009–2010
		Netmark: 2000, 2004, 2008
Household ownership of ITNs or receipt of indoor residual spraying (ITN or IRS)	The proportion of households that own at least one ITN or were sprayed with an insecticide-based solution in the last 12 months, or both	DHS: 2003, 2008, 2013
		MICS: 2011
		MIS: 2010
		LSS: 2009–2010
		Netmark: 2000, 2004, 2008
Percentage of febrile children under 5 who received ACTs	The proportion of children under 5 with a fever in the past two weeks who received artemisinin-based combination therapies (ACTs) as treatment	DHS: 2003, 2008, 2013
		MICS: 2007, 2011
		MIS: 2010
		LSS: 2009–2010
Health outcomes		
Under-5 mortality	The probability that a child born in the given year will die before reaching the age of 5; expressed in terms of under-5 deaths per 1,000 live births	DHS: 1990, 2003, 2008, 2013
		MICS: 1999, 2007, 2011
		MIS: 2010
Percentage of children under 5 who are underweight	The proportion of children under 5 who are underweight, as defined by weighing two or more standard deviations below the international anthropometric reference population median of weight for age	DHS: 2003, 2008, 2013
		MICS: 1999, 2007, 2011
Prevalence of wasting among children under 5	The proportion of children under 5 who are wasting, as defined by weighing two or more standard deviations below the international anthropometric reference population median of weight for height	DHS: 2003, 2008, 2013
		MICS: 1999, 2007, 2011
Prevalence of stunting among children under 5	The proportion of children under 5 who are stunted, as defined by weighing two or more standard deviations below the international anthropometric reference population median of height for age	DHS: 2003, 2008, 2013
		MICS: 1999, 2007, 2011

CWIQ, Core Wealth Indicator Questionnaire; DHS, Demographic and Health Survey; GHS, General Household Survey; LSS, Living Standards Survey; MICS, Multiple Indicator Cluster Survey; MIS, Malaria Indicator Survey; Netmark, Netmark Survey; SMART, Standardized Monitoring and Assessment of Relief and Transition

### Data

We conducted a comprehensive search of all available state-level survey data for Nigeria, including targeted literature reviews, indexed data files stored in the Global Health Data Exchange (GHDx) [35], and source-specific requests made to organizations and ministries. All data files extracted from the GHDx are publicly available and can be directly accessed online: http://ghdx.healthdata.org.

Surveys were excluded if they did not measure any of the study’s indicators, we could not link units of observation to a given state, or there were documented concerns about data quality or representativeness (the 1999 Demographic and Health Survey [DHS]; Community Partnership for Action in the Social Sectors [COMPASS]) [36–39]. Preference was always given to microdata, but we used tabulated state-level findings from survey reports if the underlying microdata were not available (e.g. the UNICEF Standardized Monitoring and Assessment of Relief and Transition [SMART] surveys). Nineteen surveys met inclusion criteria, as documented in Table 1.

#### MCH indicators

##### Data processing

We produced state-level estimates for MCH indicators using each data source. For this analysis, states were defined using current administrative divisions for 36 states and the Federal Capital Territory (Abuja) from Nigeria’s National Bureau of Statistics. We also used regional information to inform our analyses, as designated by each state’s geopolitical zone (North East, North Central, North West, South East, South South, South West). When microdata were available, we accounted for sampling design by incorporating provided sampling weights at this step of analysis.

All MCH indicators except for ANC1, ANC4, SBA, and IFD were extracted as prevalence estimates for the year of the survey, estimating the state-level mean and variance for a given survey-year. For ANC1, ANC4, SBA, and IFD we attributed coverage estimates to the year of birth of the child. Mothers reported information on these interventions for each child in the DHS and Core Wealth Indicator Questionnaire, allowing for the extraction six years’ worth of data from the survey date [40]. Multiple Indicator Cluster Surveys (MICS) only provided information on the mother’s most recent birth [41], so we only extracted data for births within two years of the survey date to ensure population representativeness.

In cases where data collection and measurement approaches differed across surveys, we used various standardization and cross-walking methods to generate comparable estimates of intervention coverage. For immunization indicators, some surveys only reported children’s vaccination status based on a child health card and not by both child health cards and a respondent’s self-report; in these cases, we adjusted state-level estimates of immunization coverage by applying the average relationship between immunization coverage only based on cards and total immunization coverage as determined by MICS and DHS. EBF standardization procedures are described in further detail by previous work [42].

For malaria interventions, coverage estimates of 0.01% were applied to years prior to formal policy adoption and intervention implementation by Nigeria or a given state. Coverage estimates of 0.01% were used prior to 2001 for IPTp2 [43, 44], as IPTp2 was formally included in Nigeria’s national guidelines in 2005 but may have been used during earlier years [45, 46]; prior to 2004 for ACTs [45] and prior to 2000 for ITN ownership [44]. IRS has been implemented in a phased manner in Nigeria, so we applied 0.01% coverage prior to 2000 for all states and then removed this coverage restriction in accordance with documentation of state-level IRS trials and subsequent IRS programs [6, 21–23].

Point estimates for each survey were validated by reviewing survey documentation and reports. We excluded 3% of the data due to documented sampling issues in certain states or due to implausibility relative to nearby data points.

### Estimating state-level trends

Data were synthesized using a two-stage statistical modeling approach. In the first stage we defined a mean (prior) function for each indicator using a mixed-effects model. These results were then fed into a Gaussian process regression (GPR), a Bayesian model which estimates the posterior distribution of probable trends and allows for the derivation of median estimates with uncertainty intervals. We describe the process below in general terms for all indicators.

To determine the prior mean function for each indicator, we first considered a number of possible specifications. All proposed models were either linear regression models estimated using ordinary least squares or linear mixed-effects regression models and varied in terms of what systematic and random components were included. The systematic component included a fixed effect on time. Year was included in the model either directly as a single continuous variable or represented by the bases of a natural cubic spline with a single interior knot. In the first case, the temporal pattern for the outcome was assumed to be linear while the second case allowed for a non-linear temporal pattern and was consequently more flexible [47]. Independent and identically distributed (IID) random intercepts and slopes were tested at the state level and by geopolitical zone. Dependent variables were logit-transformed to bound results between 0 and 1.

We used cross-validation to assess the predictive validity of up to 14 models for each indicator. Below is the general form of the model:

$$ {y}_{s,t}=\alpha +\mathbf{T}\left(\boldsymbol{\beta} +{\boldsymbol{b}}_s+{\boldsymbol{b}}_z\right)+{u}_s+{u}_z+{\varepsilon}_{s,t} $$

where *y*_*s*,*t*_ is the logit-transformed indicator for state *s* in year *t*; *α* is the intercept; **T** is time, represented either as linear or using a natural cubic spline; ***β*** is the vector of coefficients for time; ***b***_*s*_ is an IID random slope on time for state *s*; ***b***_*z*_ is an IID random slope on time for geopolitical zone *z*; *u*_*s*_ is an IID random intercept with mean zero for state *s*; *u*_*z*_ is an IID random intercept with mean zero for geopolitical zone *z*; *ε*_*s,t*_ is a normally distributed error term with mean zero for state *s* in year *t*.

For each indicator, we selected the model with the lowest root-mean squared error as derived from a 20% random hold-out pattern repeated 100 times.

The median posterior trend and uncertainty were estimated using GPR for each indicator and state. GPR has been used for similar cross-country and subnational modelling applications and is explained in detail elsewhere [2, 40, 48–50]. We used the estimates generated in the first stage regression as the mean function with a covariance structure defined by the Matern covariance function. We used 1,000 random draws from the posterior distribution to calculate median trends and confidence intervals (CIs), with the latter represented by the 25th and 975th ordered draws. Finally, national-level trends for each indicator were estimated by population-weighting state estimates at the draw level.

#### Under-5 mortality

To estimate trends in under-5 mortality for each state in Nigeria we applied data processing and synthesis methods developed previously [51]. Briefly, we extracted one-year summary and complete birth history data from multiple surveys (DHS, MICS, and Malaria Indicators Surveys [MIS]) to estimate source year-specific probabilities of death before the age of 5 years [52]. We then modeled under-5 mortality trends by applying a one-knot natural spline model with state and survey IID random effects. We then population-weighted state-level estimates to generate a national trend for under-5 mortality [53]. We systematically compared this national trend with Nigeria-specific results from the Global Burden of Disease 2013 study to derive an under-5 mortality scaling factor [2]. This scaling factor was then applied to state-level estimates.

### Overall intervention coverage

We constructed an overall intervention coverage metric to examine levels and trends across multiple key MCH indicators that reflect the priorities of Nigeria’s health system. This metric included 11 interventions: three malaria interventions (household ownership of ITNs and/or IRS, IPTp2 coverage, and receipt of ACTs among febrile children), four childhood vaccines (BCG, measles, OPV3, and DPT3), and four other MCH indicators (ANC4, SBA, EBF, and the proportion of children under 5 who were not wasted). Each indicator was equally weighted for the overall intervention coverage metric, which was based on the average of the 11 indicators.

### Ethical approval

Ethical approval for this study was obtained from the institutional review board of the University of Washington. The study was conducted in compliance with national regulatory and ethics guidelines. Any personal identifiers collected during survey administration were removed before household surveys were made publicly available; as a result, we only analyzed de-identified survey data.

## Results

### Health outcomes

#### Under-5 mortality

**Fig. 1:**
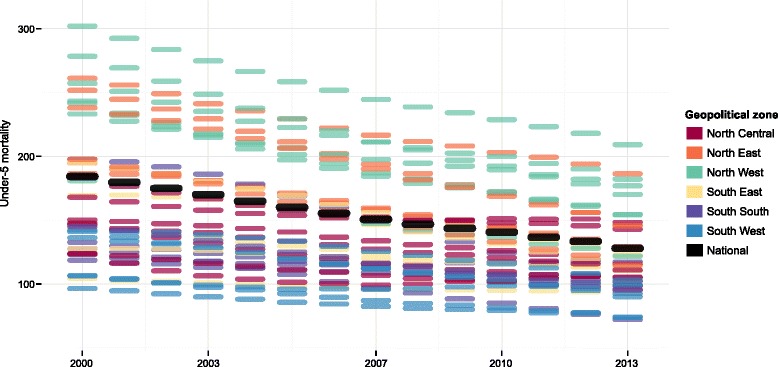
Trends in under-5 mortality by state, 2000–2013. Note: Each line represents a state grouped by geopolitical zone. The black line represents the national trend for under-5 mortality

Nationally, under-5 mortality decreased by 30% from 2000 to 2013, declining from 184 deaths per 1,000 live births (95% CI, 178–191) in 2000 to 128 deaths per 1,000 live births (95% CI, 114–142) in 2013 (Fig. 1). Under-5 mortality rates fell in every state, with the range between states narrowing from 2000 to 2013. In 2000, the state-level difference in under-5 mortality was 206, ranging from 96 deaths per 1,000 live births in Lagos (95% CI, 82–114) to 302 deaths per 1,000 live births in Zamfara (95% CI, 258–349). By 2013, this difference shrunk to 137, ranging from 72 deaths per 1,000 live births in Edo (95% CI, 52–95) to 209 deaths per 1,000 live births in Zamfara (95% CI, 160–269). Despite large gains in under-5 survival, Zamfara consistently has had the highest state-level under-5 mortality in Nigeria since 2000.

**Fig. 2:**
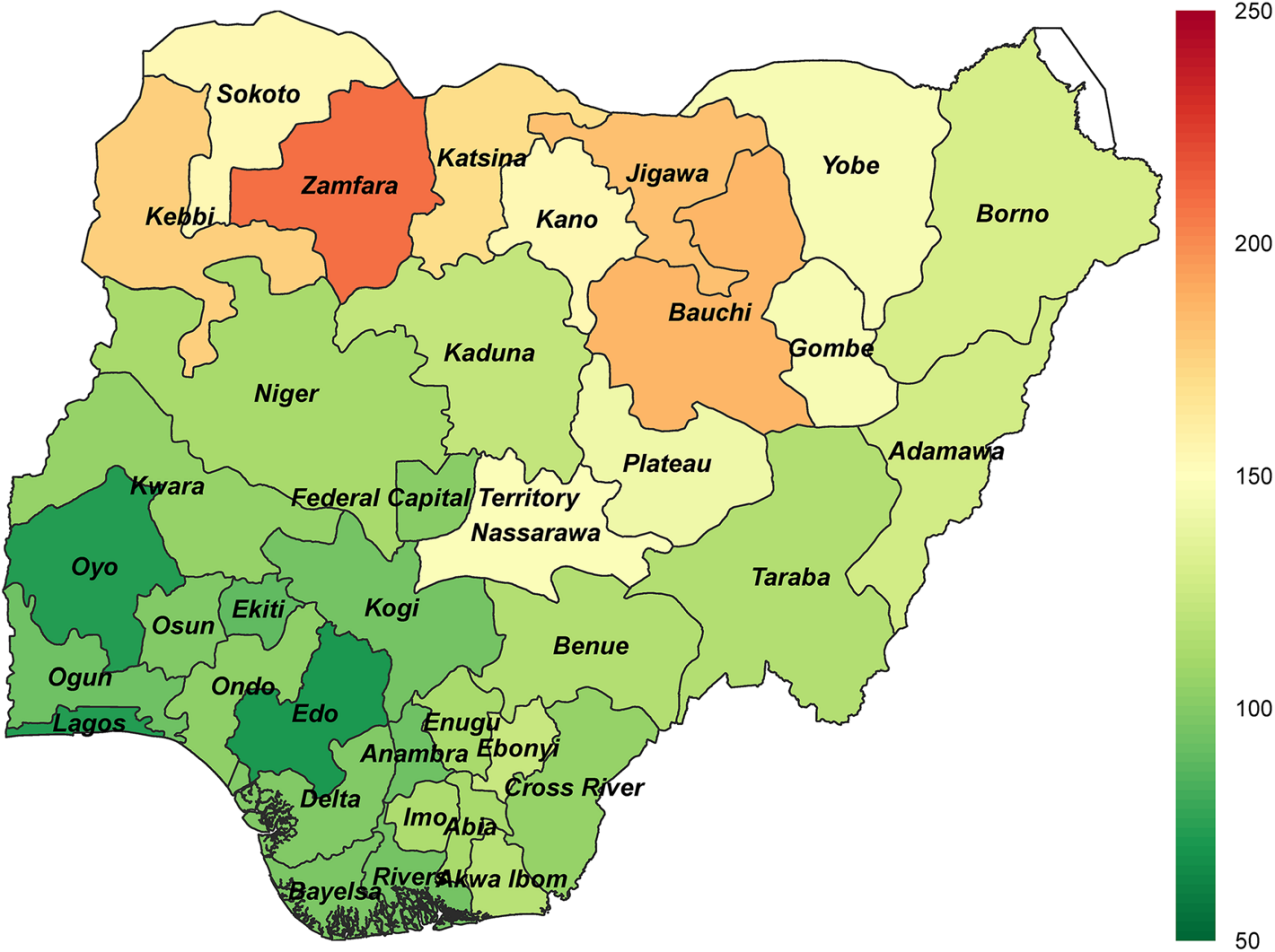
Under-5 mortality by state in 2013

Figure 1 shows that, for the most part, states with higher under-5 mortality in 2000 recorded the largest declines by 2013. Nevertheless, regional patterns of inequality persisted, with states in the North West and North East zones still experiencing much higher rates of under-5 mortality in 2013 than those located in southern areas (Fig. 2). In 2013, seven northern states had under-5 mortality rates exceeding 150 deaths per 1,000 live births, similar to national rates experienced by countries with the highest levels of under-5 mortality worldwide (Guinea Bissau [153 deaths per 1,000 live births], Mali [149 deaths per 1,000 live births], and Chad [147 deaths per 1,000 live births]) [2]. In contrast, under-5 mortality in 2013 was around 70 deaths per 1,000 live births in Edo, Lagos, and Oyo. While these states had the lowest levels of under-5 mortality in Nigeria in 2013, they still remained twice as high as rates recorded by several other sub-Saharan African countries (e.g. Botswana [31 deaths per 1,000 live births] and Namibia [35 deaths per 1,000 live births]) [2].

#### Child nutrition

**Fig. 3:**
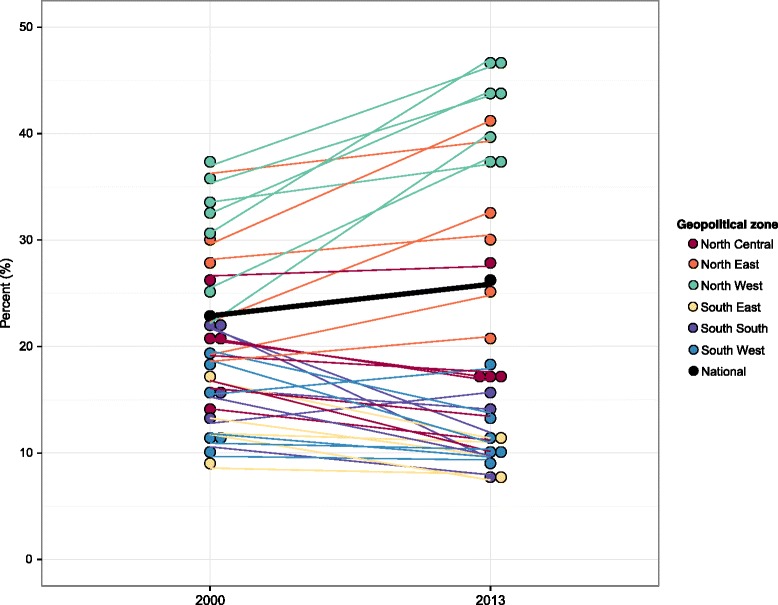
Percentage of children under 5 who are underweight by state in 2000 and 2013

Our results showed a small, though not statistically significant, increase in the proportion of children who were underweight in Nigeria, from 23% (95% CI, 21–25%) in 2000 to 26% (95% CI, 24–28%) in 2013. At the state level, results were mixed in terms of decreasing and rising rates of childhood underweight from 2000 to 2013, though for most states the change was not statistically significant during this time (Fig. 3). States with high prevalence of underweight children in 2000, primarily in the North West and North East zones of the country, saw increases in prevalence while those with the lowest levels in 2000 experienced moderate progress. Of note, Kaduna and Cross River states had very similar rates of childhood underweight in 2000, but Kaduna state experienced one of the largest increases in prevalence between 2000 and 2013, while Cross River state showed the largest decrease. These trends point to widening inequalities across Nigeria, as rates of underweight children ranged from 7% in Enugu (95% CI, 6–9%) to 47% in Kebbi (95% CI, 33–62%) in 2013.

Other indicators of child nutrition pointed to signs of moderate progress at the national level, though these changes also were not statistically significant. For instance, at the national level, the proportion of children who were stunted declined from 39% in 2000 (95% CI, 37–41%) to 35% in 2013 (95% CI, 33–37%), and rates of wasting decreased from 18% (95% CI, 16–20%) to 16% (95% CI, 14–17%) during this time. Across states, regional patterns more closely followed those found for rates of childhood underweight, with trends implying rising prevalence of stunting in some northern states. The prevalence of wasting remained fairly unchanged over time across states, although some recorded moderate declines in wasting (Additional file 2).

### Interventions

#### Malaria control

**Fig. 4:**
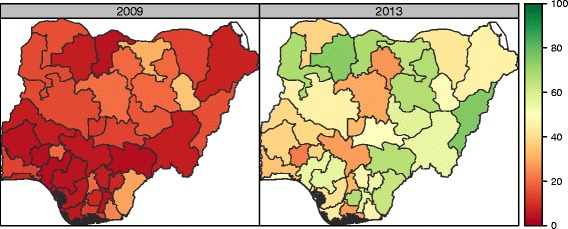
Household ownership of insecticide-treated nets or receipt of indoor residual spraying, or both, by state in 2009 and 2013

Nationally, ITN ownership increased from near zero in the early 2000s to 48% in 2013 (95% CI, 41–55%). Most of these gains were driven by rising levels of ITN coverage occurred after 2009, when a number of state-level ITN distribution campaigns began. Across states, ITN or IRS ranged from 3% in Benue (95% CI, 1–13%) to 35% in Gombe (95% CI, 6–82%) in 2009. By 2013, the range in coverage widened, spanning from 23% in Osun (95% CI, 5–63%) to 75% in Adamawa (95% CI, 21–97%; Fig. 4). The geographic patterns for ITN or IRS coverage varied substantially compared to the trends observed for other indicators, such that ITN or IRS coverage was generally much higher in areas with lower levels of non-malaria interventions.

IPTp2 coverage remained low over time, only reaching 20% nationally in 2013 (95% CI, 15–25%) and ranging from 4% in Edo (95% CI, 0–27%) to 48% in Niger (95% CI, 18–82%). Nigeria implemented ACTs as the first-line treatment against uncomplicated malaria in 2005 [45], yet the receipt of ACTs among children under 5 with suspected malaria also remained very low in 2013, at 9% nationally (95% CI, 6–13%). All states had rates of ACT coverage below 25% in 2013, with Rivers recording the highest at 20% (95% CI, 4–57%). Additional file 2 provides more results for these malaria interventions.

No one state consistently had high levels of coverage across all malaria interventions; instead, most of the states with higher levels of ITN or IRS coverage recorded relatively lower rates of IPTp2 and ACT coverage, and vice versa.

#### Childhood immunizations

Vaccination rates for measles, DPT3, and OPV3 increased in Nigeria from 2000 to 2013; however, Nigeria’s absolute levels of coverage remained low with no vaccine exceeding 65% coverage nationally. Coverage trends varied substantially by geographic region and vaccine.

Nationally, measles immunization coverage rose from 44% in 2000 (95% CI, 41–46%) to 55% in 2013 (95% CI, 52–57%), though state-level vaccination rates ranged from 8% in Sokoto (95% CI, 3–18%) to 92% in Ekiti (95% CI, 86–96%) that year. While most states experienced increases in measles immunization coverage between 2000 and 2013, 14 states recorded declines in coverage after 2005. Oyo had one of the most notable drops in measles immunization, falling from 78% in 2005 (95% CI, 70–85%) to 68% in 2013 (95% CI, 58–80%). More results on measles immunization coverage can be found in Additional file 2.

**Fig. 5:**
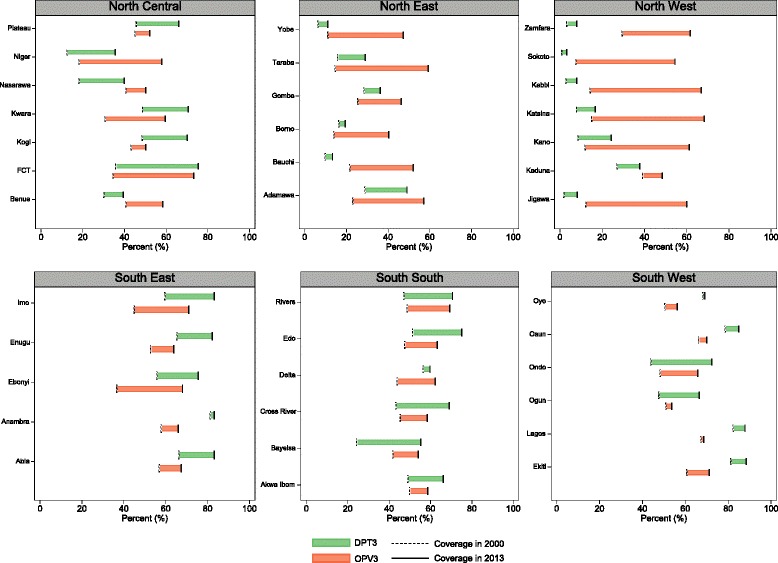
Changes in OPV3 and DPT3 immunization coverage by state from 2000 to 2013

State-level trends in coverage for DPT3 and OPV3, both vaccines with similar dosing requirements and immunization schedules [54, 55], were particularly heterogeneous. Figure 5 shows coverage rates of DPT3 and OPV3 for every state, grouped by geopolitical zone, for 2000 and 2013. Increases in OPV3 coverage were particularly pronounced for several states in North West and North East, which traditionally have been high-priority targets for Nigeria’s polio elimination campaigns [56]. Increased DPT3 coverage lagged behind the gains recorded for the receipt of OPV3. For instance, in Kebbi and Katsina, the difference between coverage of OPV3 and DPT3 exceeded 50 percentage points. By contrast, many states in the North Central and South South zones recorded similar increases in OPV3 and DPT3 coverage over time. In a number of southern states, DPT3 immunization coverage exceeded OPV3 vaccination rates. In Lagos state, DPT3 coverage was 19 percentage points higher than OPV3 coverage, with 88% DPT3 coverage (95% CI, 76–94%) and 69% OPV3 coverage (95% CI, 48–83%). Further, state-level differences in coverage varied for these two vaccines. In 2013, DPT3 immunization rates spanned from 3% in Sokoto (95% CI, 1–9%) to 88% in Ekiti (95% CI, 78–94%) and 88% in Lagos (95% CI, 76–94%). Conversely, the gap narrowed between states with the highest and lowest levels of OPV3 coverage over time, decreasing from a difference of 59 percentage points in 2000 and 33 percentage points in 2013.

#### Other key MCH interventions

For more routine MCH services, Nigeria largely experienced minimal progress or declines in coverage between 2000 and 2013; however, most decreases in coverage were not statistically significant. Nationally, ANC coverage somewhat decreased since 2000, slipping to 71% for ANC1 (95% CI, 69–72%) and 61% for ANC4 (95% CI, 59–62%) by 2013. Coverage of SBA and IFD was typically lower than ANC indicators, particularly in the North East and North West zones. Nationally, EBF coverage was 15% in 2013 (95% CI, 13–18%), well below Nigeria’s EBF target of 38% for 2013 [10]. Across states, coverage inequalities remained high for ANC, IFD, and SBA. For instance, we found a difference of 86 percentage points between the state with the highest levels of IFD in 2013 (Imo, at 91% [95% CI, 87–95%]) and the state with the lowest (Zamfara, at 5% [95% CI, 2–12%]).

**Fig. 6:**
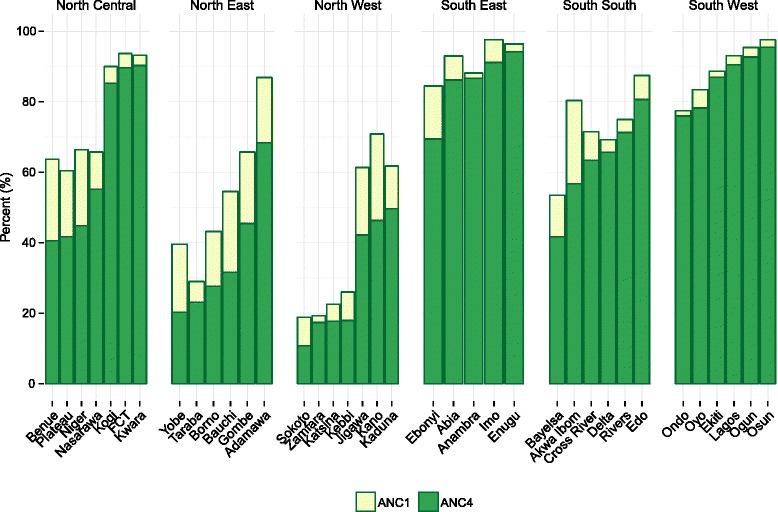
ANC1 and ANC4 coverage by state in 2013

Several states also experienced potential gaps in the continuum of care for maternal health services. One example is the gap in coverage between ANC1 and ANC4 (Fig. 6), which may reflect challenges in ensuring that pregnant women receive the recommended four ANC visits [10, 57]. For instance, Kano’s coverage of ANC1 was 71% in 2013 (95% CI, 63–78%), but its rates of ANC4 were much lower, at 47% (95% CI, 38–56%). By contrast, this difference was much lower for many states in the South East zone. Ondo recorded rates of ANC1 and ANC4 at 77% (95% CI, 69–84%) and 76% (95% CI, 67–84%), respectively. Over time, the relationship between ANC1 and ANC4 varied widely across states. Many states, such as Kwara and Enugu, saw coverage of ANC4 become closer to ANC1 since 2000, whereas others saw coverage gaps widen. Several states experienced declines in both ANC1 and ANC4 coverage, but with the latter falling faster, emphasizing the importance of monitoring various indicators along continuums of care.

### Overall intervention coverage

**Fig. 7:**
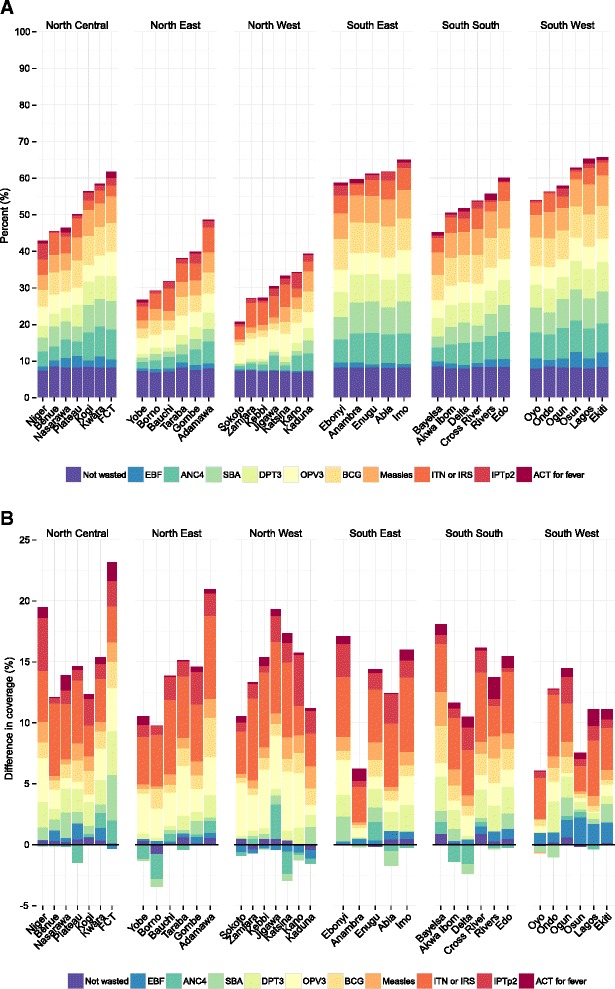
Overall intervention coverage in 2013 (**a**) and change in overall intervention coverage from 2000 to 2013 (**b**). Note: The relative contribution of each individual intervention is shown within each state bar. In (**b**), bar height represents the absolute change, in percentage points, for overall intervention coverage from 2000 to 2013

Based on 11 high-priority MCH interventions in Nigeria, we found that overall intervention coverage varied drastically across states and within geopolitical zones (Fig. 7a). States in the North West zone had some of the country’s lowest levels of overall intervention coverage in 2013, ranging from 21% in Sokoto to 39% in Kaduna. Among these states, coverage was high for a subset of interventions (ITN ownership or IRS and OPV3 immunization); for other interventions, however, coverage remained low. Eight states, largely located in the South East and South South zones, and the Federal Capital Territory had overall intervention coverage equaling or exceeding 60% in 2013, with Ekiti recording the highest level of coverage (66%). For these states, the relative contribution of each intervention was more balanced across MCH indicators. Figure 7a clearly shows that EBF was the intervention with consistently low levels of coverage across all states in Nigeria.

Overall intervention coverage increased for all states between 2000 and 2013, but how each intervention contributed to – or detracted from – these gains varied across Nigeria (Fig. 7b). Rising coverage of ITNs or IRS was the main driver of improved overall intervention coverage for most states. Increasing immunization rates also contributed to this progress, particularly in the North West zone. At the same time, several states had marked declines in coverage for ANC4 and SBA, which hindered further state-level gains in overall intervention coverage. A number of states, largely in the North West zone, also experienced rising rates of wasting among children under 5. Overall growth among states varied from approximately 5 to 23 percentage points, reflecting the diverse nature of Nigeria’s state-level health landscapes.

## Discussion

This study represents the first-ever assessment of state-level trends for a range of MCH interventions and outcomes in Nigeria, highlighting the country’s mixture of progress and ongoing challenges in improving local health service provision. Every state recorded declines in under-5 mortality – a major success – yet, absolute rates of child deaths still ranked among the highest in sub-Saharan Africa [2]. For a subset of malaria interventions, coverage increased substantially between 2009 and 2013; however, coverage remained quite low, particularly for IPTp2 and ACTs, a cause for concern given Nigeria’s large malaria burden [4]. Coverage of certain immunizations increased, especially for OPV3, an important result given Nigeria’s aims to end wild polio transmission [56]. At the same time, similar gains were not actualized for DPT3 coverage across states, suggesting that more routine delivery platforms for multi-dose vaccines may be faltering amid disease-focused immunization campaigns [40, 58]. Coverage of other key MCH interventions, such as ANC4 and SBA, generally stagnated or declined, and stark differences in coverage have persisted across states since 2000. Benchmarking state-level performance for MCH indicators demonstrated the continued entrenchment of North-South differences, particularly for more routine services, and showed that overall intervention coverage generally remained low despite recent gains for a subset of MCH interventions.

Nigeria’s state-level trends in under-5 mortality likely reflect the complex nature of improving health outcomes amid local changes in health system access, delivery of services, broader socio-economic gains, and overall development. While all states experienced reductions in under-5 mortality between 2000 and 2013, it is unlikely that these gains were driven by the same factors in each state. In states where malaria transmission is particularly high and coverage of ITNs increased, such as Bauchi, improved childhood survival may be attributable to expanded malaria control programming. In contrast, in urban areas where malaria transmission is somewhat lower and women’s educational attainment rose since 2000, such as Edo, socio-economic advances may play a stronger role in reducing under-5 deaths. Assessing how different intervention packages and socio-economic forces are contributing the largest gains in childhood survival – and doing so at local levels – is critical to accelerating improved health throughout Nigeria. For disease-specific programs that feature well-funded, focused campaigns (namely malaria and polio) [5–7], the scale-up of intervention coverage was less varied across states. For instance, by 2013, many states in more rural, poor areas reached levels of OPV3 coverage found in the wealthier states of Nigeria. Although absolute levels of immunization coverage remained lower than optimal, the gap between states with the highest and lowest levels of coverage narrowed over time. Conversely, inequalities in the coverage of several MCH interventions and services provided through more routine platforms continued across states, often following geographic patterns for urbanicity, wealth, and educational attainment [32, 59]. These findings suggest that barriers to accessing and using health services likely remain in many states, particularly those in more remote, impoverished areas. These factors involve ongoing violence in northern Nigeria [60–62]; demand-side influences (proximity to facilities and care [63–65], affordability of transportation to health facilities [66], cost of health care [67], knowledge of available services and trust in providers [28], religious views or cultural mores [68, 69]); and supply-side dynamics (availability of skilled medical staff and their interactions with patients [70–72], inconsistent stocks of pharmaceuticals and medical supplies across levels of care [73–75], inadequate facility infrastructure [76]). In combination, these factors may have a compounding effect on hindering health-care-seeking behaviors. Additional work on identifying which of these factors are most easily affected by policy levers and programs promoting heighted utilization of routine services in Nigeria should be prioritized.

Nigeria’s gains in improving polio immunization coverage, particularly when compared to trends in DPT3 vaccination rates, highlight the differences in the country’s health system functions across specific interventions. As one of the last polio-endemic countries in the world, Nigeria has received tremendous resources and policy attention for eliminating polio, especially through the GPEI [56, 77]. Due to targeted immunization campaigns and developing disease surveillance structures [56, 78, 79], Nigeria increased OPV3 immunization coverage by 25 percentage points since 2000 and created a strong detection-response system for finding its remaining polio cases. Such progress is particularly impressive given that Nigeria has experienced tensions and violence around polio vaccination, notably culminating in boycotts of immunization campaigns in Kano, Zamfara, and Kaduna in 2003 and 2004 [80, 81], and multiple shootings in 2013 [82]. On the contrary, Nigeria saw minimal progress for DPT3 immunization rates, another three-dose vaccine that is delivered through more routine EPI services rather than mass campaigns. This contrast with Nigeria’s gains in OPV3 coverage reflects how program-focused investments and commitment can likely improve vaccination rates, as well as possible missed opportunities for integrating service delivery [55, 58].

Diverging state-level trends for MCH interventions revealed geographic disparities along a continuum of care for maternal health services. In a health system where both demand and supply for health services are strong, we might expect to see similar coverage levels for ANC1, ANC4, SBA, and IFD, or that women have least four ANC visits prior to delivery and give birth in a health facility and/or in the presence of skilled attendant [83]. We found that a subset of states, largely located in the southern regions of Nigeria, appeared to have strong linkages between these services, experiencing minimal differences in coverage for ANC1 and ANC4, for example. However, the majority of states saw some kind of breakdown in this MCH service continuum. These findings point to two related but separate challenges to bolstering Nigeria’s continuum of care for MCH services: (1) improving the frequency of ANC visits and (2) cultivating stronger demand and capacity for giving birth with skilled birth attendants or in health facilities. Previous research has identified cost, transportation, and capacity of the health facility as barriers for women seeking ANC services [84, 85] and SBA [14, 86, 87]. Local health authorities may consider expanding and scaling up existing programs which include outreach campaigns, improving facility-based resources for ANC and routine deliveries, and innovative incentive structures, such as conditional cash transfers, that explicitly link ANC to post-natal services [1, 14, 16, 88–91].

This study further demonstrates the importance of setting ambitious yet realistic health system goals, as well as approaching such target-setting with an equity lens. Nigeria established several high-reaching health program goals to improve MCH outcomes, which included achieving 80% ITN coverage by 2013 [92], 78% DPT3 coverage by 2013 [54], and rates of 38% for EBF by 2013 [10]. We found that national coverage of these interventions – 47% for ITN ownership, 46% for DPT3, and 15% for EBF – registered well below the country’s targets in 2013. While a few states met or exceeded these targets for EBF and DPT3 (e.g., Osun and Ekiti had EBF rates exceeding 40% in 2013), most states fell quite short of the country’s health program goals. Many of these targets may have been overly ambitious given baseline levels of intervention coverage (e.g., at 14% ITN ownership in 2009, Nigeria remained 66 percentage points away from its goal of 80% in 2013); at the same time, Nigeria’s goals align with global recommendations and targets for improving priority MCH outcomes (e.g. the MDGs). To accelerate Nigeria’s progress toward its health system goals for 2015 and beyond, a heightened focus on the country’s most disadvantaged populations will be required. EBF, for example, is considered highly cost-effective for improving childhood survival, requires minimal investments in health system infrastructure to scale up, and has been expanded rapidly in other African countries [42]; thus, strengthening educational outreach about EBF [93] and expanding facility-based breastfeeding programs, such as the Baby Friendly Hospital Initiative [94], across levels of care may promote better child health outcomes in states with low levels of EBF. Without a greater focus on local health needs and addressing Nigeria’s persistent health inequalities, the gaps between states with the highest and lowest levels of intervention coverage will likely widen over time. This finding also emphasizes the need for incorporating explicit equity goals within the next generation of international target-setting with the Sustainable Development Goals [95].

Our findings correspond with recent policy developments in Nigeria, namely the National Health Bill’s enactment in December 2014 [17]. This bill aims to address many of the MCH indicators analyzed in our study, with leadership claiming that its effective implementation will save over 3 million “lives of mothers, newborns, and under-5’s by 2022” [96]. The National Health Bill’s success hinges upon successful execution throughout Nigeria, a feat that has challenged the country’s past health reforms [11, 12, 97]. The use of subnational benchmarking to monitor indicators related to the National Health Bill will be critical for tracking local progress, promptly identifying obstacles in implementation, and building local accountability mechanisms. These efforts may be further improved by strengthening local health information systems, namely Nigeria’s District Health Information System (DHIS2) [98], and expanding the types of indicators captured by these systems (e.g. data pertaining to non-communicable diseases).

### Limitations

Our findings need to be interpreted within the context of some study limitations. First, we were unable to estimate trends for a number of priority MCH indicators due to data scarcity. For instance, we were unable to generate intervention estimates for HIV/AIDS treatment or case management of pneumonia as they were not captured by data sources that met inclusion criteria. Estimates of state-level maternal deaths also could not be appropriately generated due to small numbers. Second, our results do not reflect the quality of interventions received, which is a critical input for understanding whether interventions are effectively provided and thus result in their intended health gains. Third, most indicators were based on self-reports from survey respondents, and thus may be prone to various self-report biases. Fourth, our results provide minimal information about supply-side factors that affect health service provision, such as facility stocks of pharmaceuticals and medical supplies, human resources for health, and facility infrastructure. With the expansion of DHIS2 and recent release of Nigeria’s MDG Information System [33], it is possible that future analyses may account for such supply-side influences. Fifth, our findings were limited to state-level analyses due to geographic data restrictions. In the future, it would be ideal to track health trends at the LGA- or ward-level and stratified by wealth quintiles to provide more localized, actionable results. Finally, our study was descriptive in nature, and thus could not provide insights into the causes of gains, declines, and differences in state-level performance over time. Evaluating the underlying drivers of these changes over time and across states would provide invaluable insights into which types of programs work – and which do not – to improve health outcomes.

## Conclusions

With this study we implemented a systematic framework to harness available data and generate comparable trends of priority MCH outcomes and interventions over time. We found that Nigeria made notable gains for a subset of MCH indicators between 2000 and 2013, but also experienced stalled progress and even declines for others. Despite progress for a subset of indicators, Nigeria’s absolute levels of intervention coverage remained quite low. As Nigeria rolls out its National Health Bill and seeks to strengthen its delivery of health services, continued monitoring of local health trends will help policymakers track successes and promptly address challenges as they arise. Subnational benchmarking ought to occur regularly in Nigeria and throughout sub-Saharan Africa to inform local decision-making and bolster health system performance.

## Additional files

Additional file 1:
**Key maternal and child health indicators by state in 2000 and 2013*.** *Malaria indicators are shown for 2009 and 2013. (DOCX 6053 kb) Additional file 2:
**Results for key maternal and child health indicators from 2000 to 2013, state and national.** (XLSX 383 kb)

## Abbreviations

ACT: Artemisinin-based combination therapy
ANC: Antenatal care
BCG: Bacillus Calmette-Guérin
CI: Confidence interval
DHIS2: District Health Information System
DHS: Demographic and Health Survey
DPT3: Diphtheria-pertussis-tetanus vaccine, three doses
EBF: Exclusive breastfeeding
GHDx: Global Health Data Exchange
GPEI: Global Polio Eradication Initiative
GPR: Gaussian process regression
IID: Independent and identically distributed
IFD: In-facility deliveries
IPTp2: Intermittent preventive therapy during pregnancy, two doses
IRS: Indoor residual spraying
ITN: Insecticide-treated net
LGA: Local government area
MCH: Maternal and child health
MDG: Millennium Development Goal
OPV3: Oral polio vaccine, three doses
SBA: Skilled birth attendance
