# Supplementary material for: Benchmarking health system performance across states in Nigeria: a systematic analysis of levels and trends in key maternal and child health interventions and outcomes, 2000–2013
Source: BMC Med. 2015 Sep 2;13:208. doi: 10.1186/s12916-015-0438-9 (PMC4557921; doi:10.1186/s12916-015-0438-9)
Supplement: Additional file 1: — Key maternal and child health indicators by state in 2000 and 2013*. *Malaria indicators are shown for 2009 and 2013. (DOCX 6053 kb) [file 12916_2015_438_MOESM1_ESM.docx]

**Additional file 1: Key MCH indicators by state in 2000 and 2013***

*Malaria indicators are shown for 2009 and 2013

***Health outcomes***

**Under-5 mortality**

**
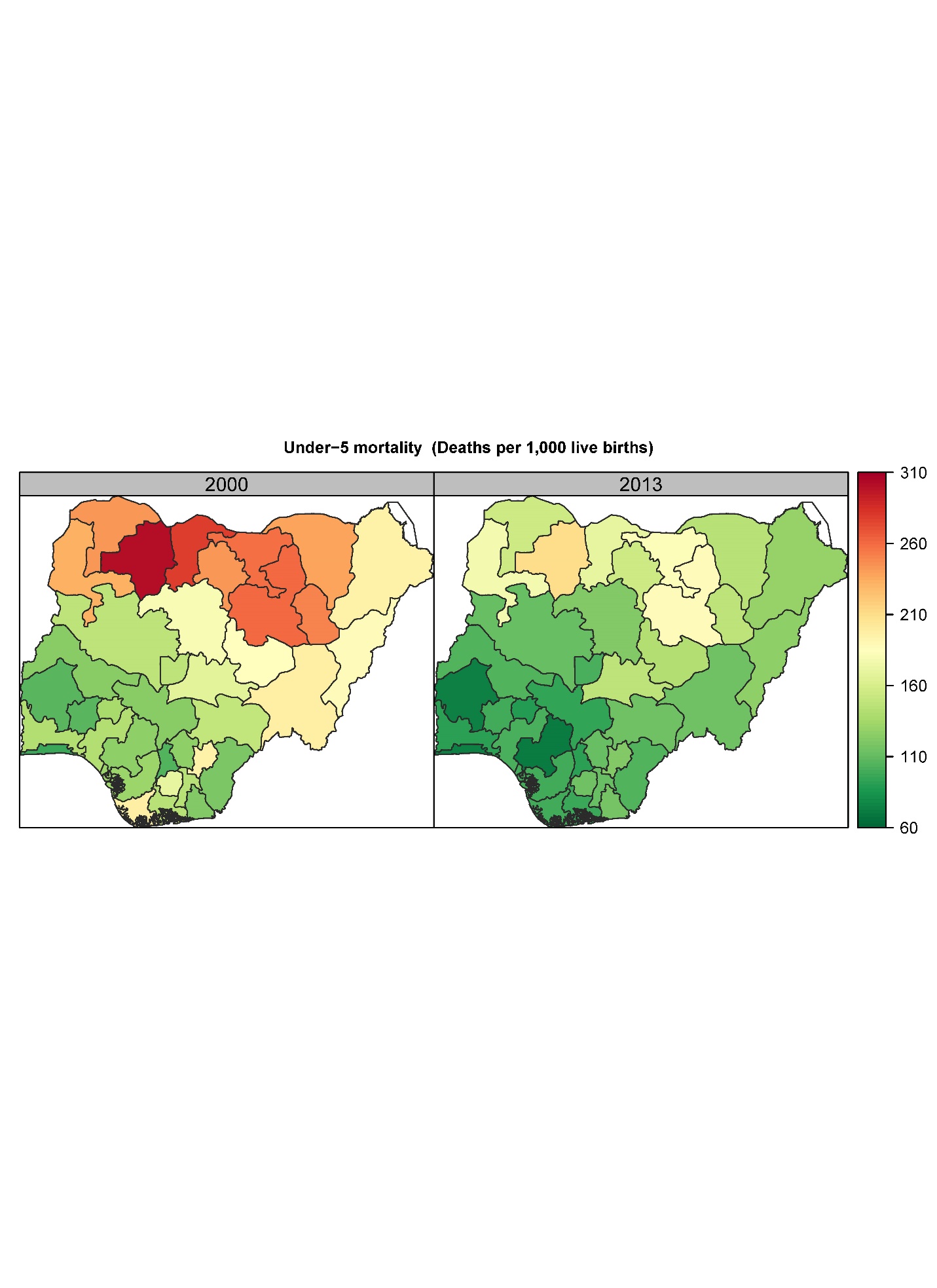
**

**Percentage of children under 5 who are underweight**

**
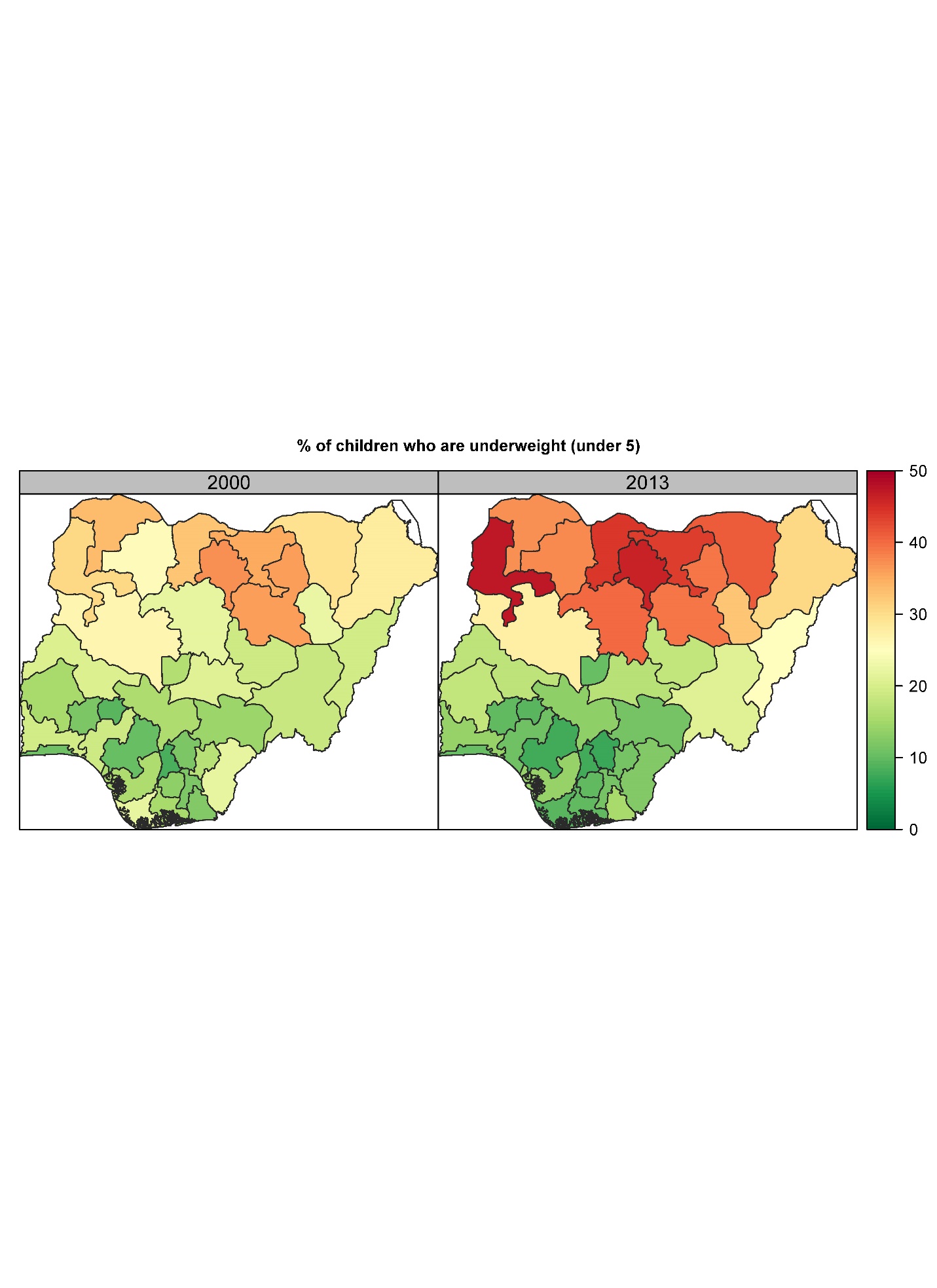
**

**Prevalence of wasting among children under 5**

**
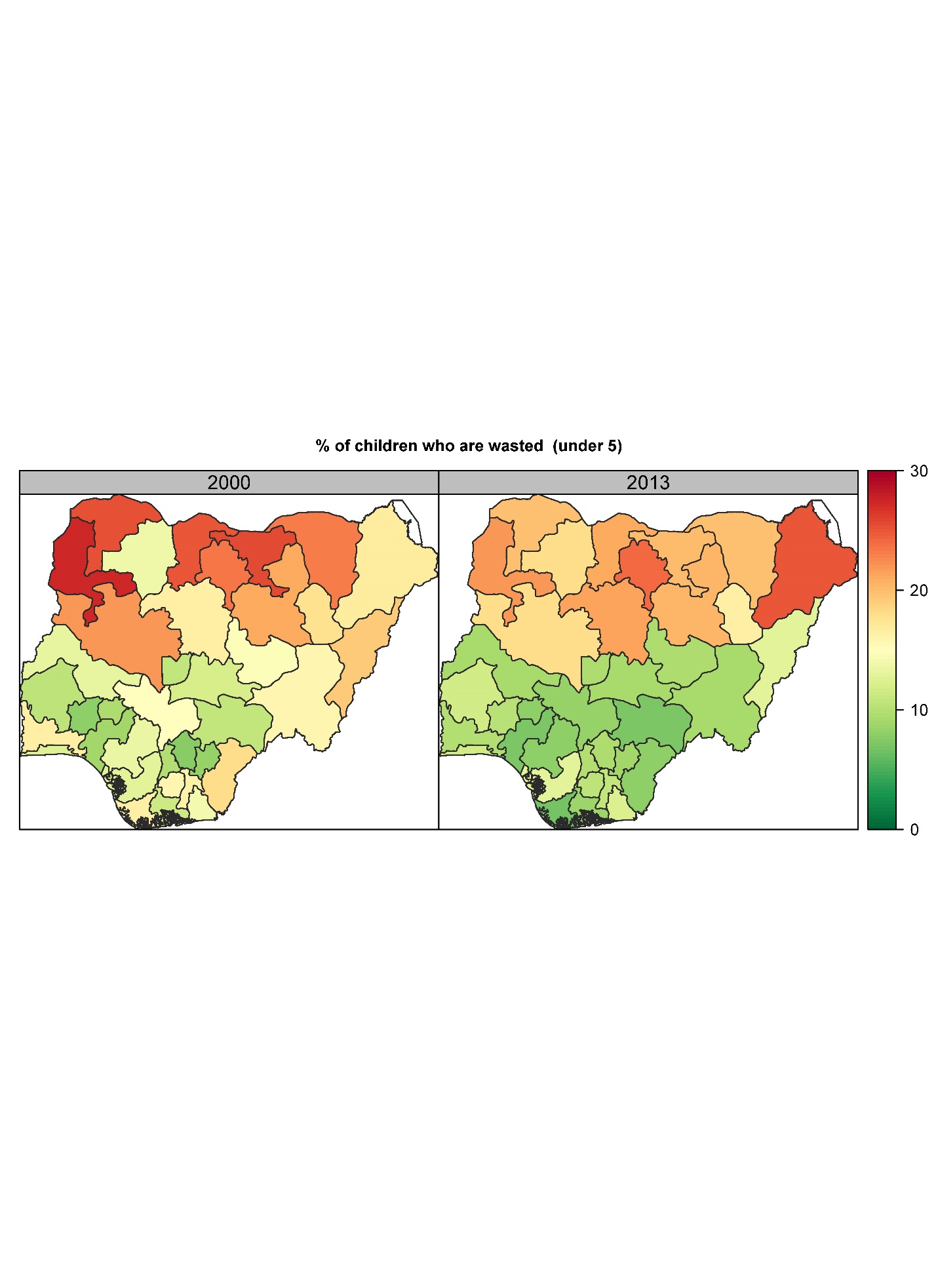
**

**Prevalence of stunting among children under 5**

**
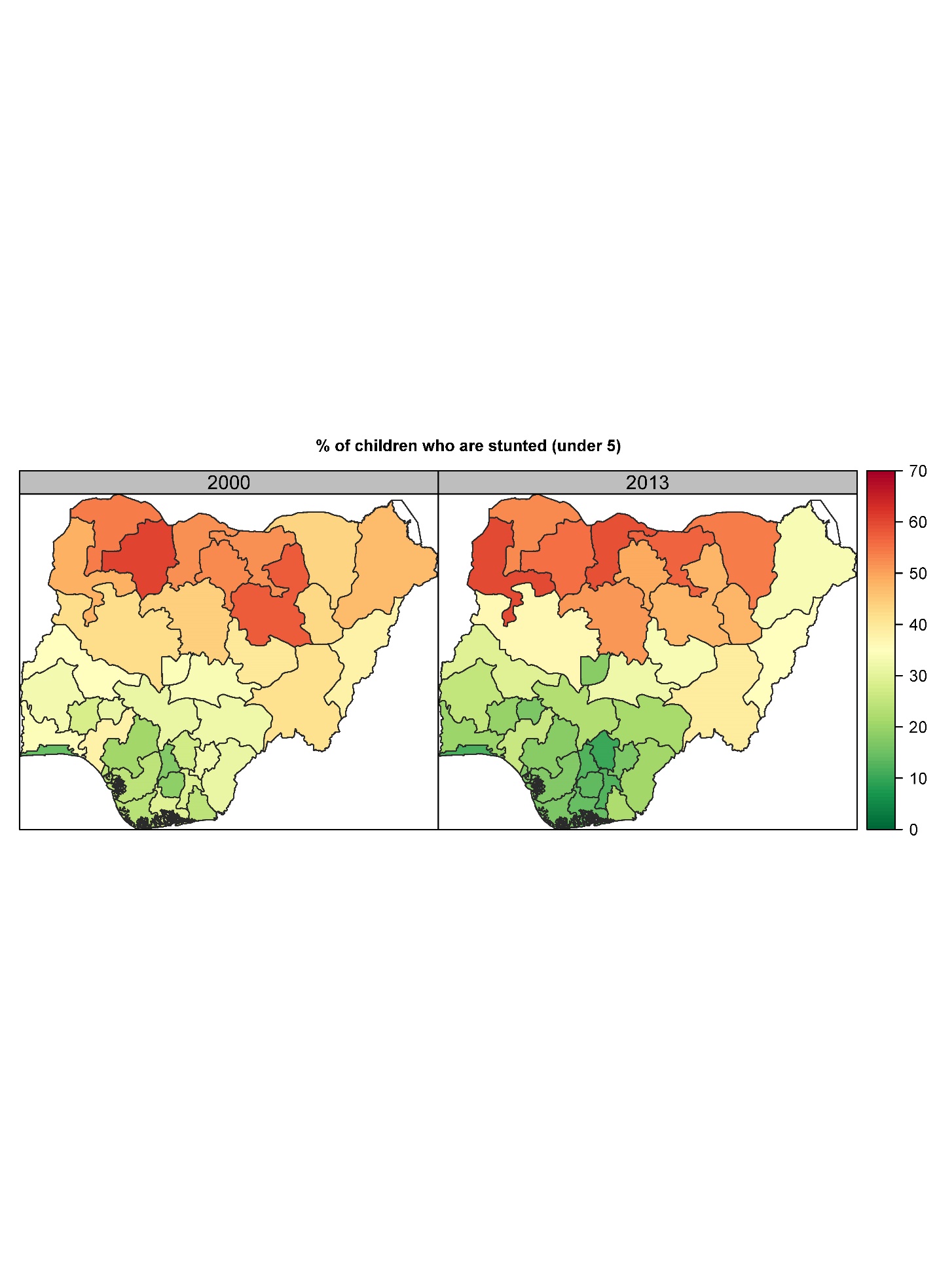
**

***Malaria interventions***

**Household ownership of at least one ITN**

**
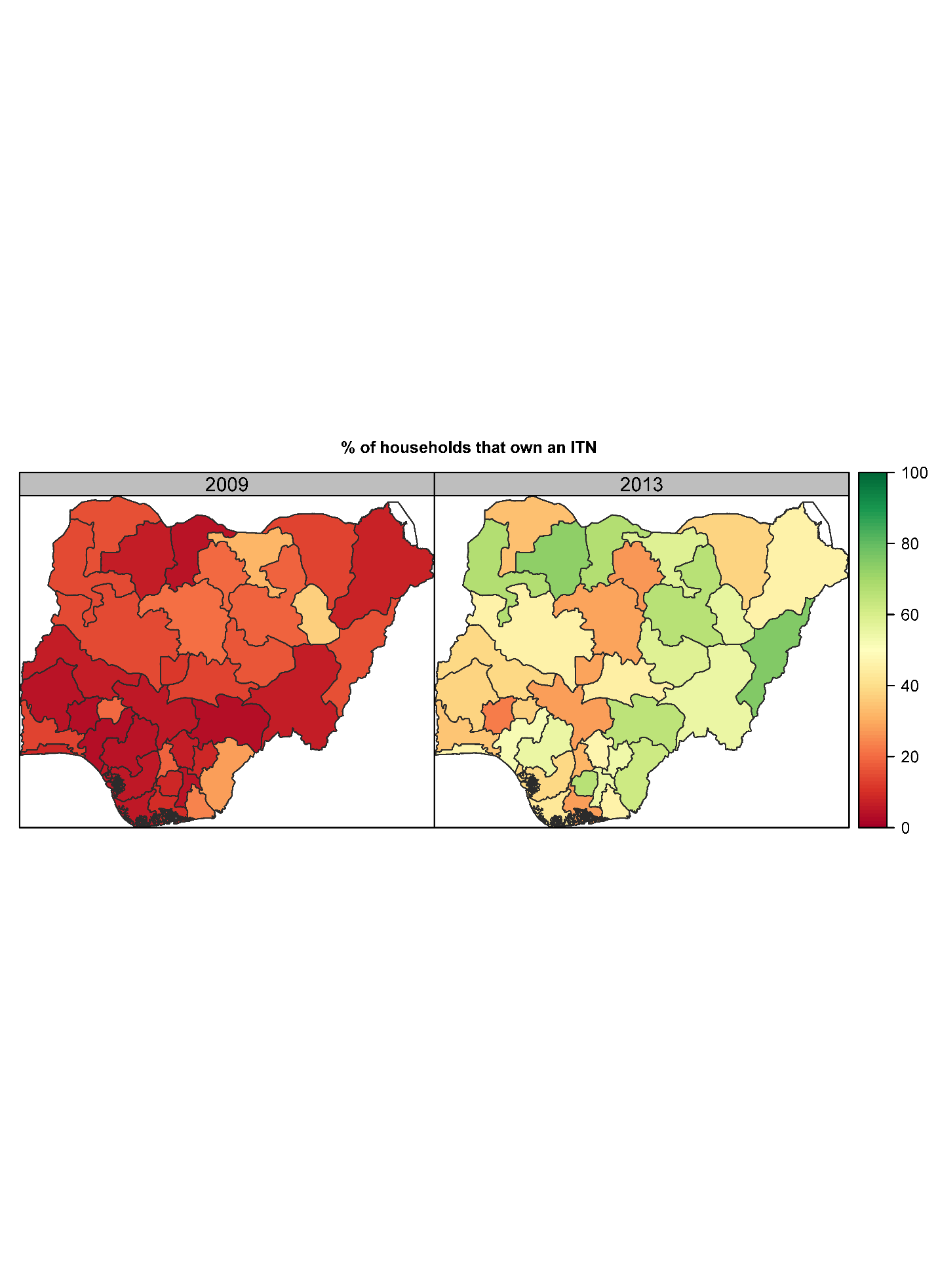
**

**Household ownership of at least one ITN or receipt of IRS, or both**


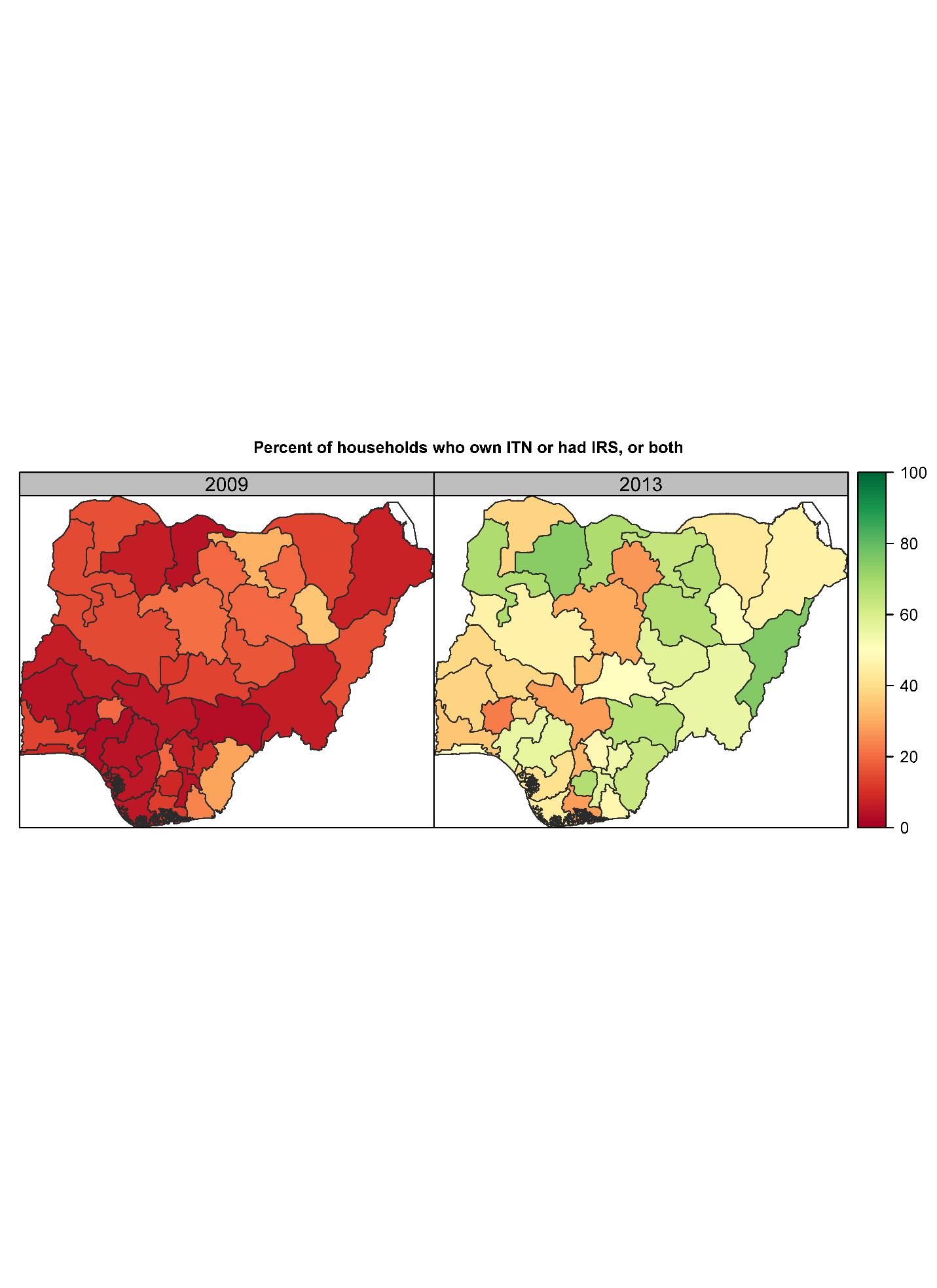


**ITN use by children under 5**

**
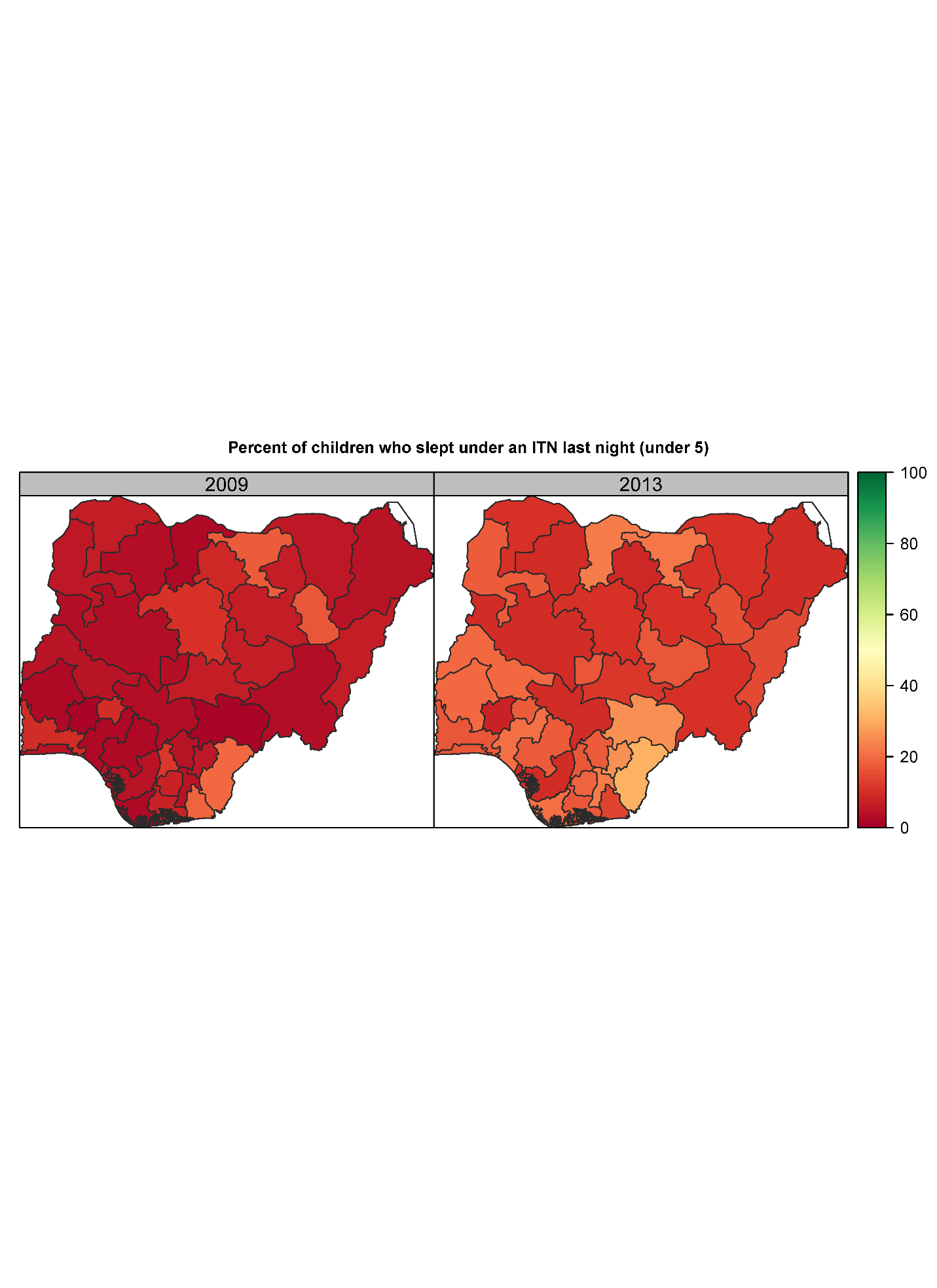
**

**Percentage of children under 5 who received ACTs for having a fever during the last two weeks**

**
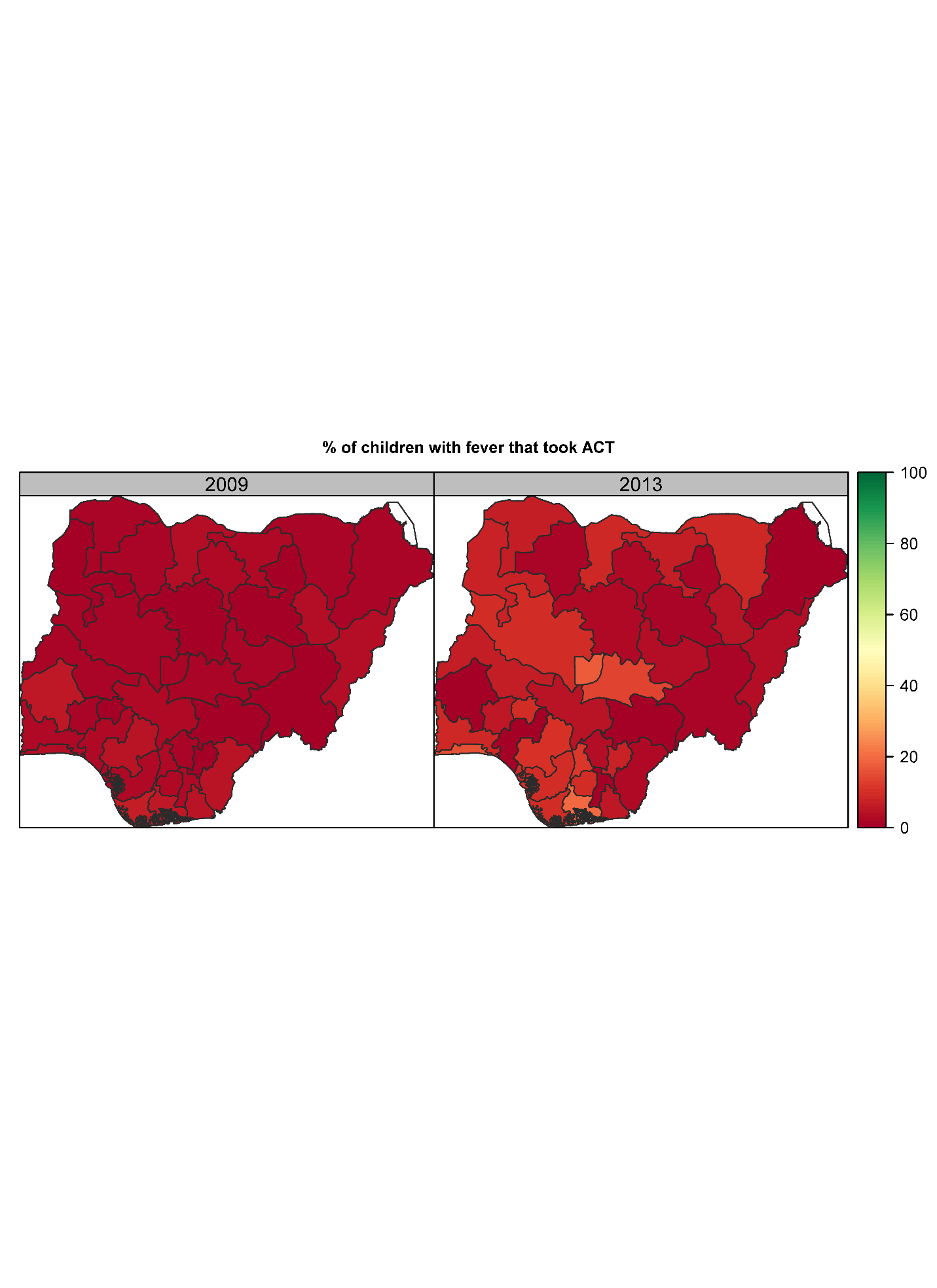
**

**Intermittent preventive therapy during pregnancy, two doses (IPTp2)**

**
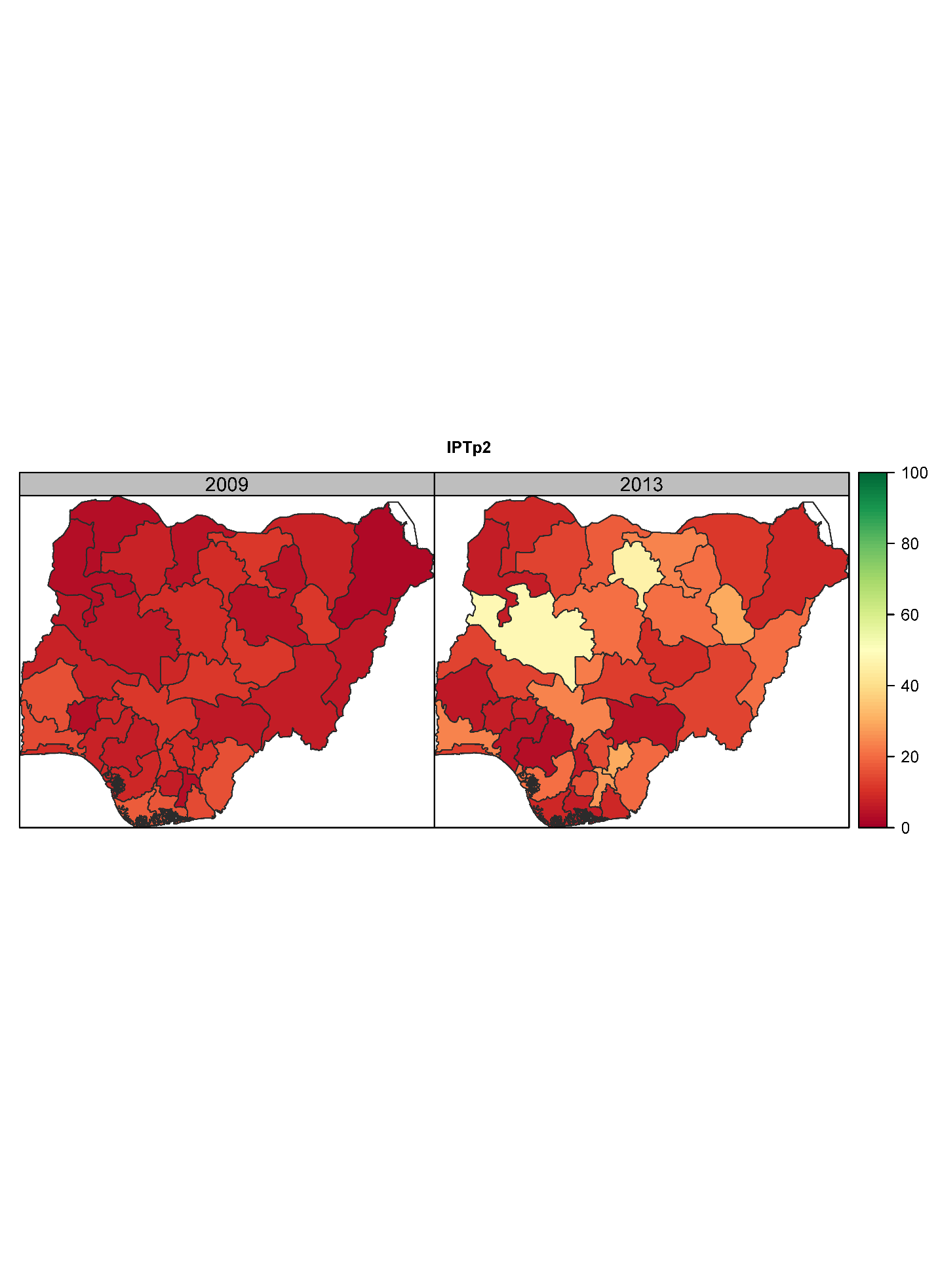
**

***Childhood immunizations***

**BCG immunization**

**
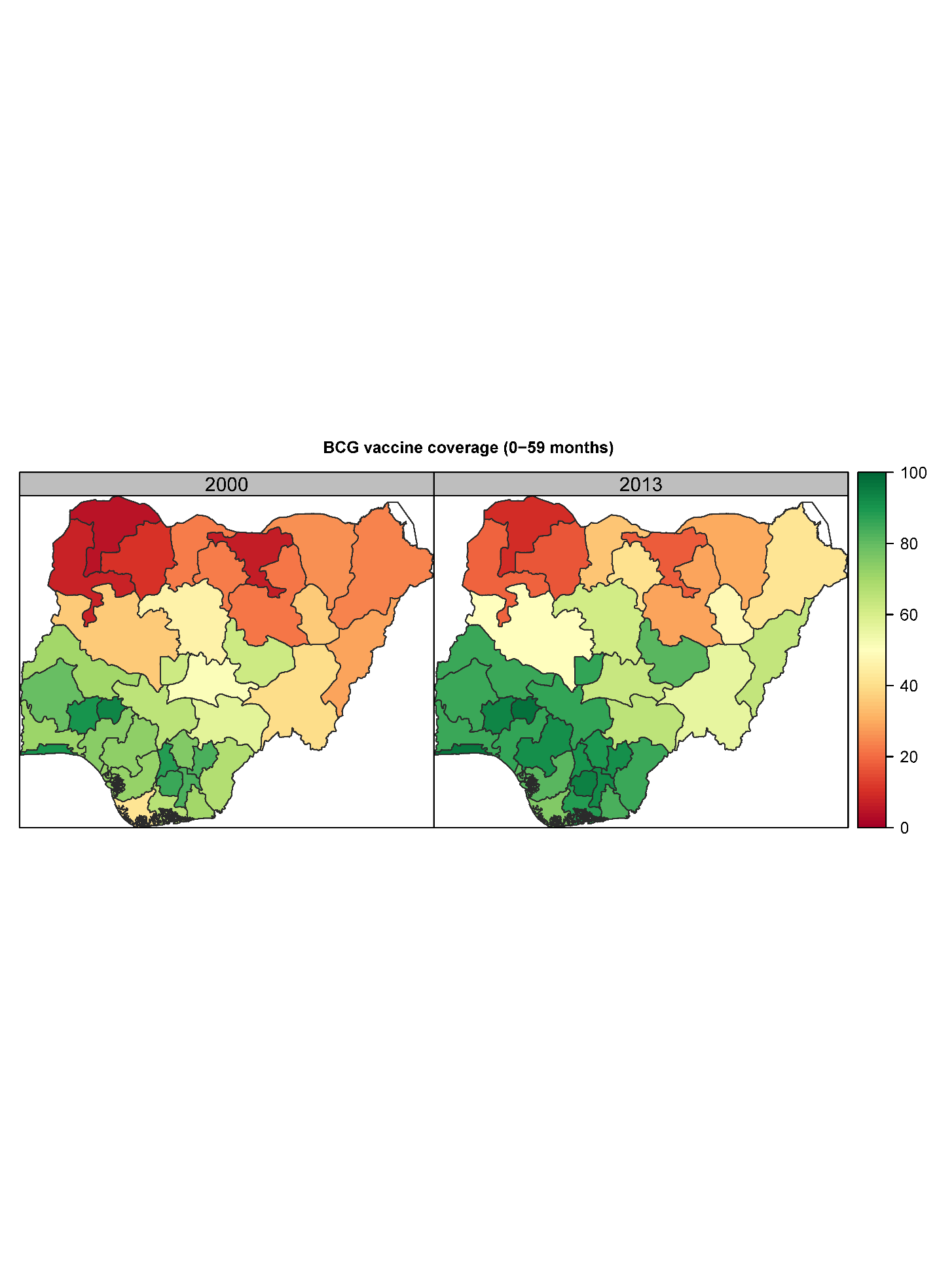
**

**Measles immunization**

**
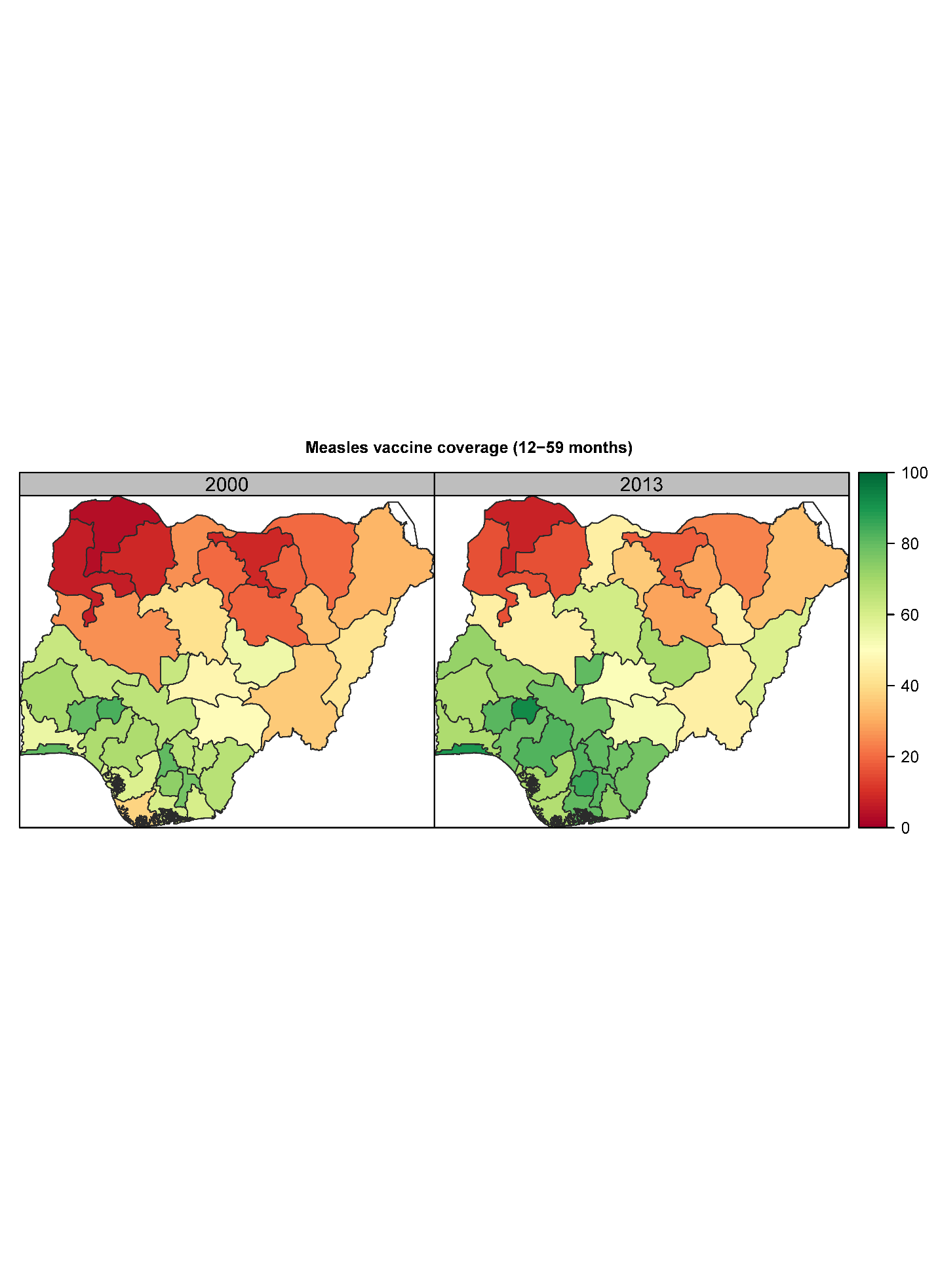
**

**DPT3 immunization**

**
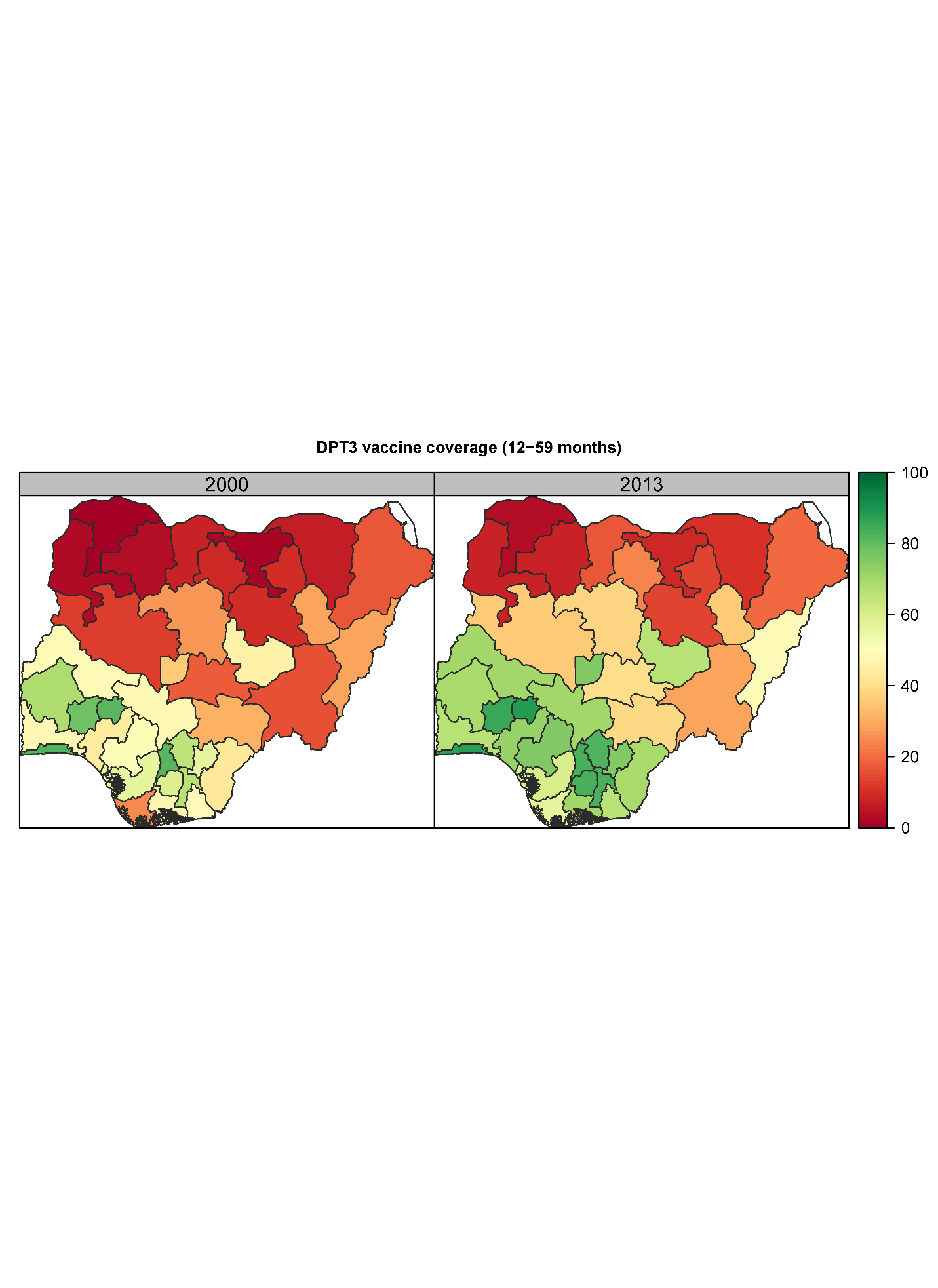
**

**OPV3 immunization**

**
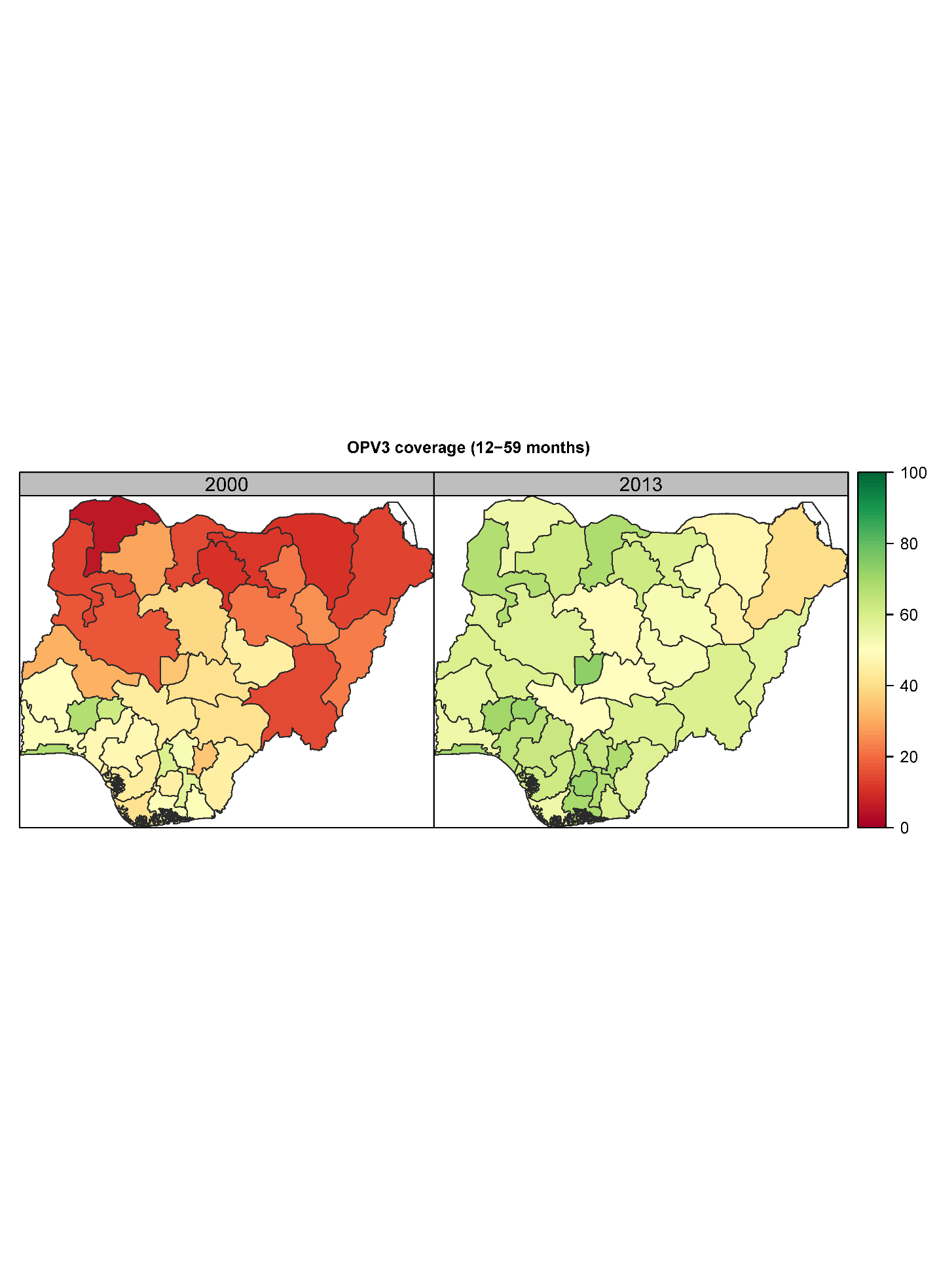
**

***Other key MCH interventions***

**Antenatal care, 1 visit (ANC1)**

***
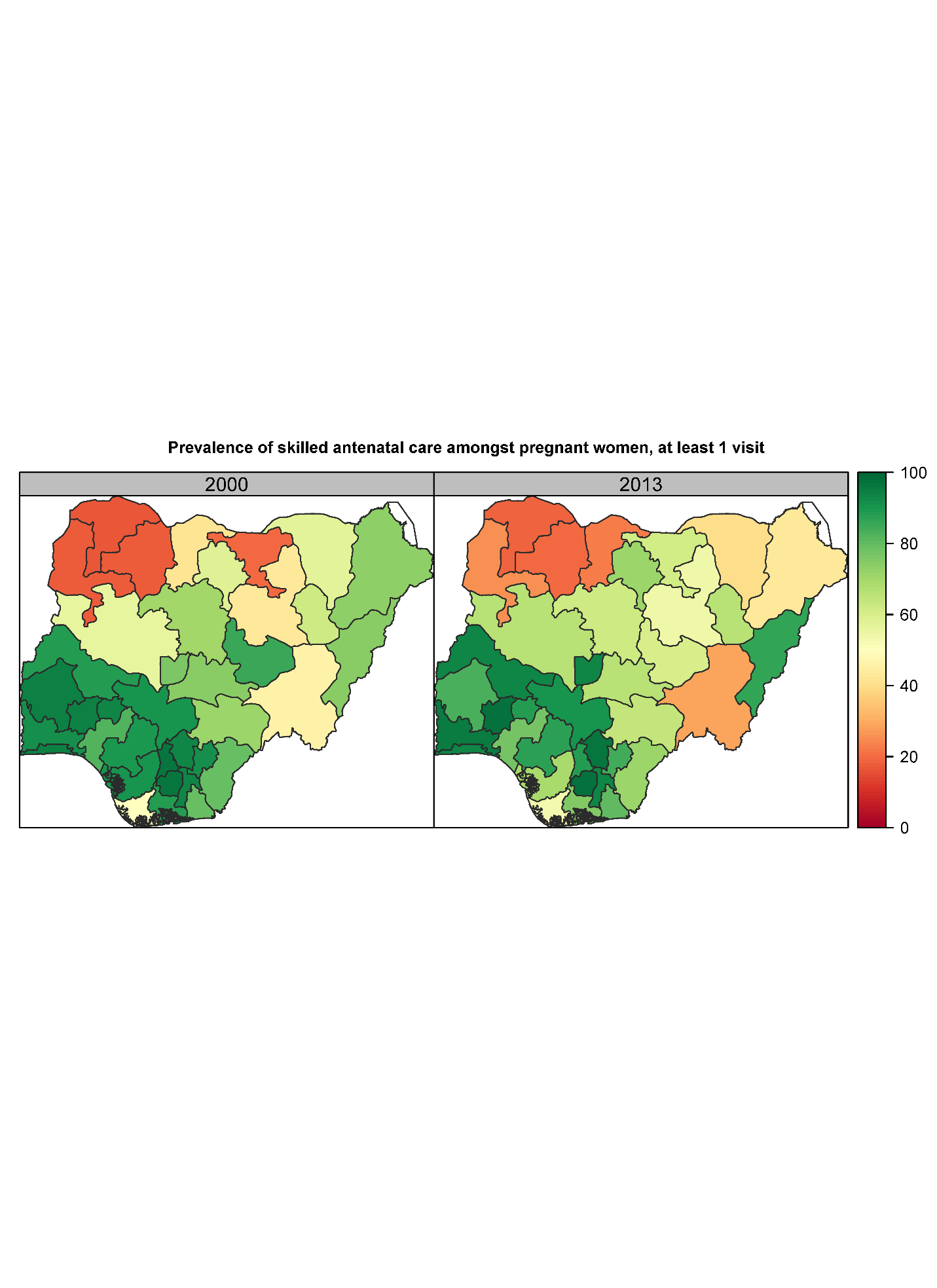
***

**Antenatal care, 4 visits (ANC4)**

**
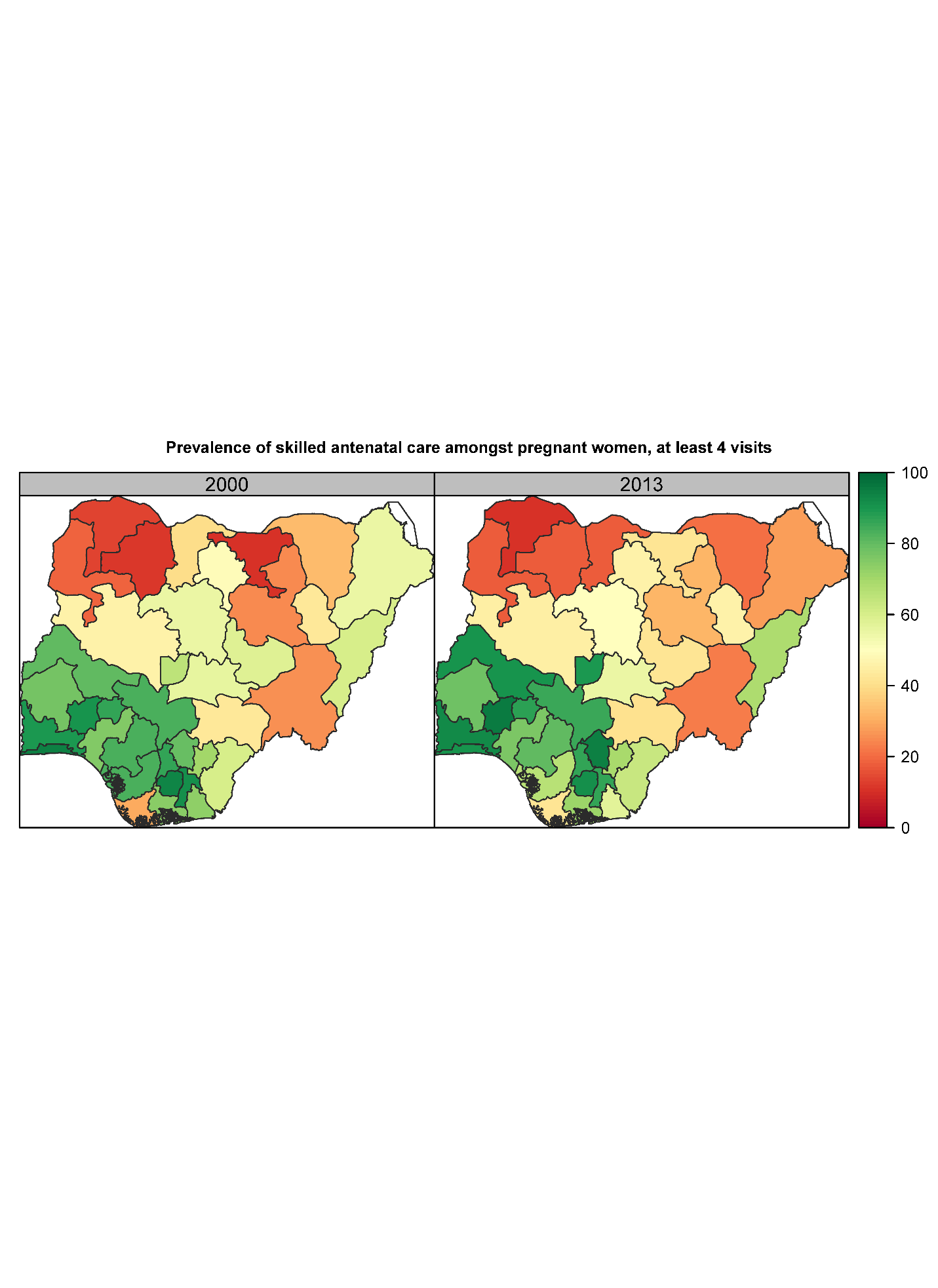
**

**Skilled birth attendance**

**
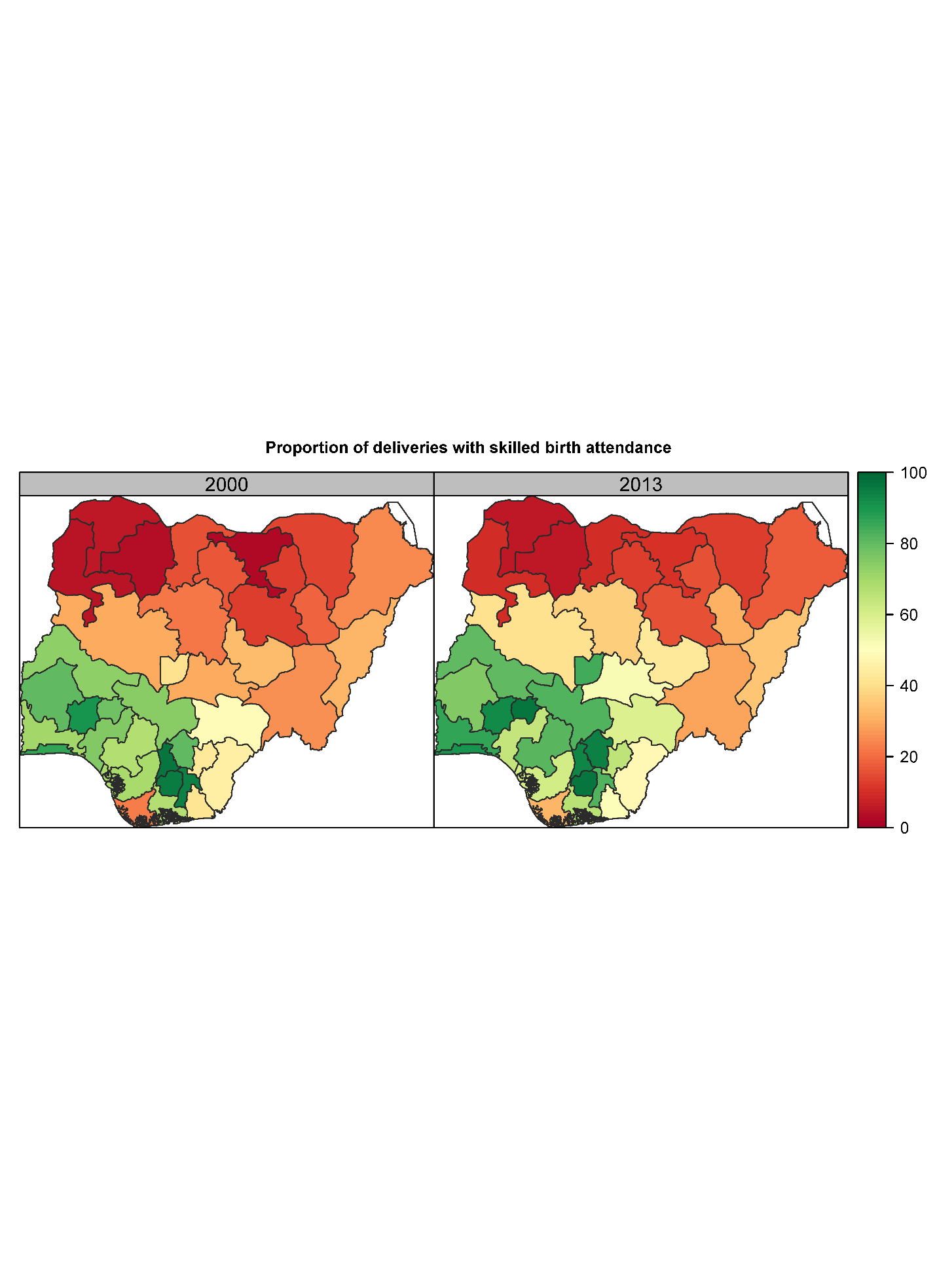
**

**In-facility delivery rate**

**
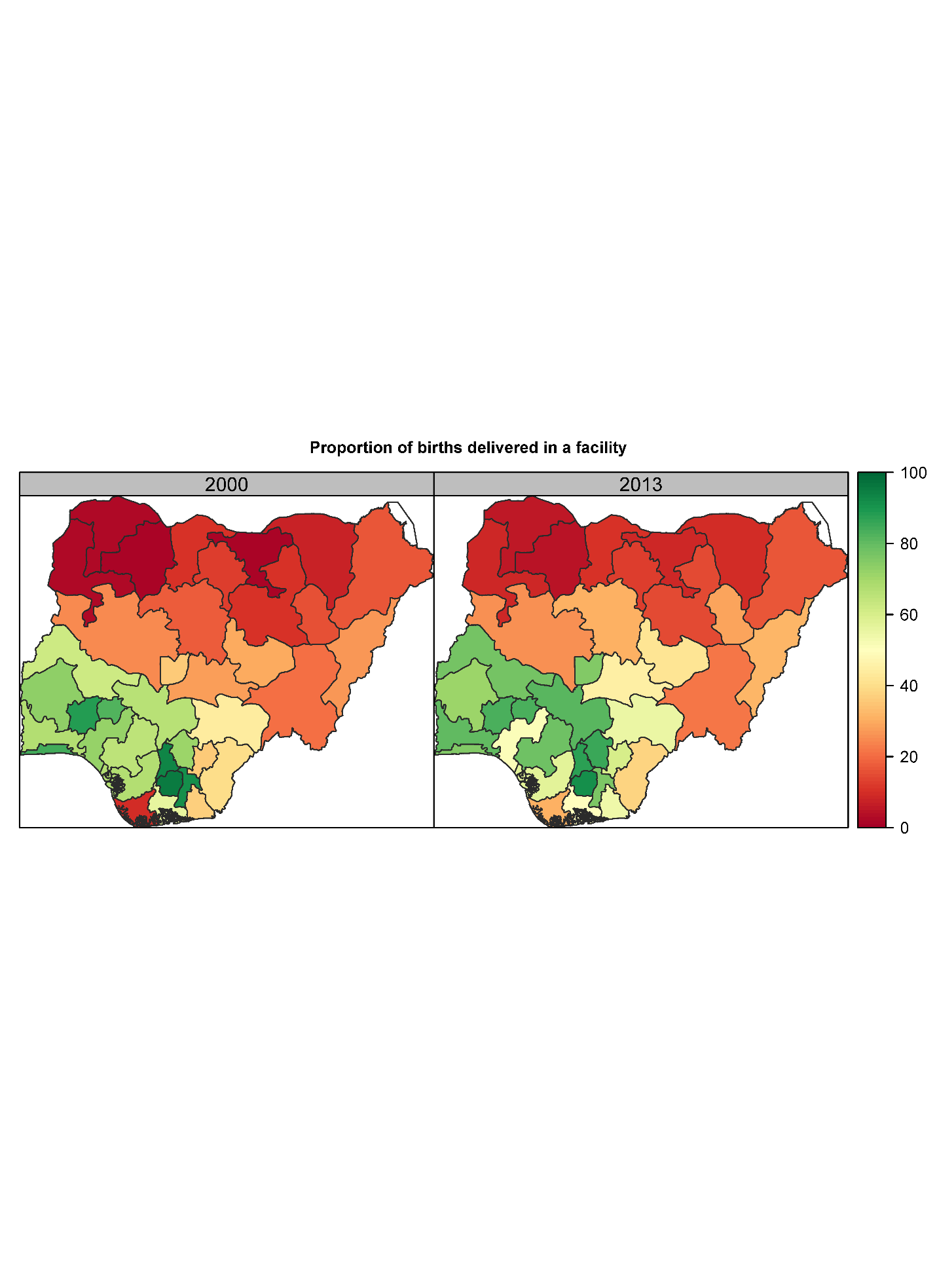
**

**Exclusive breastfeeding**

**
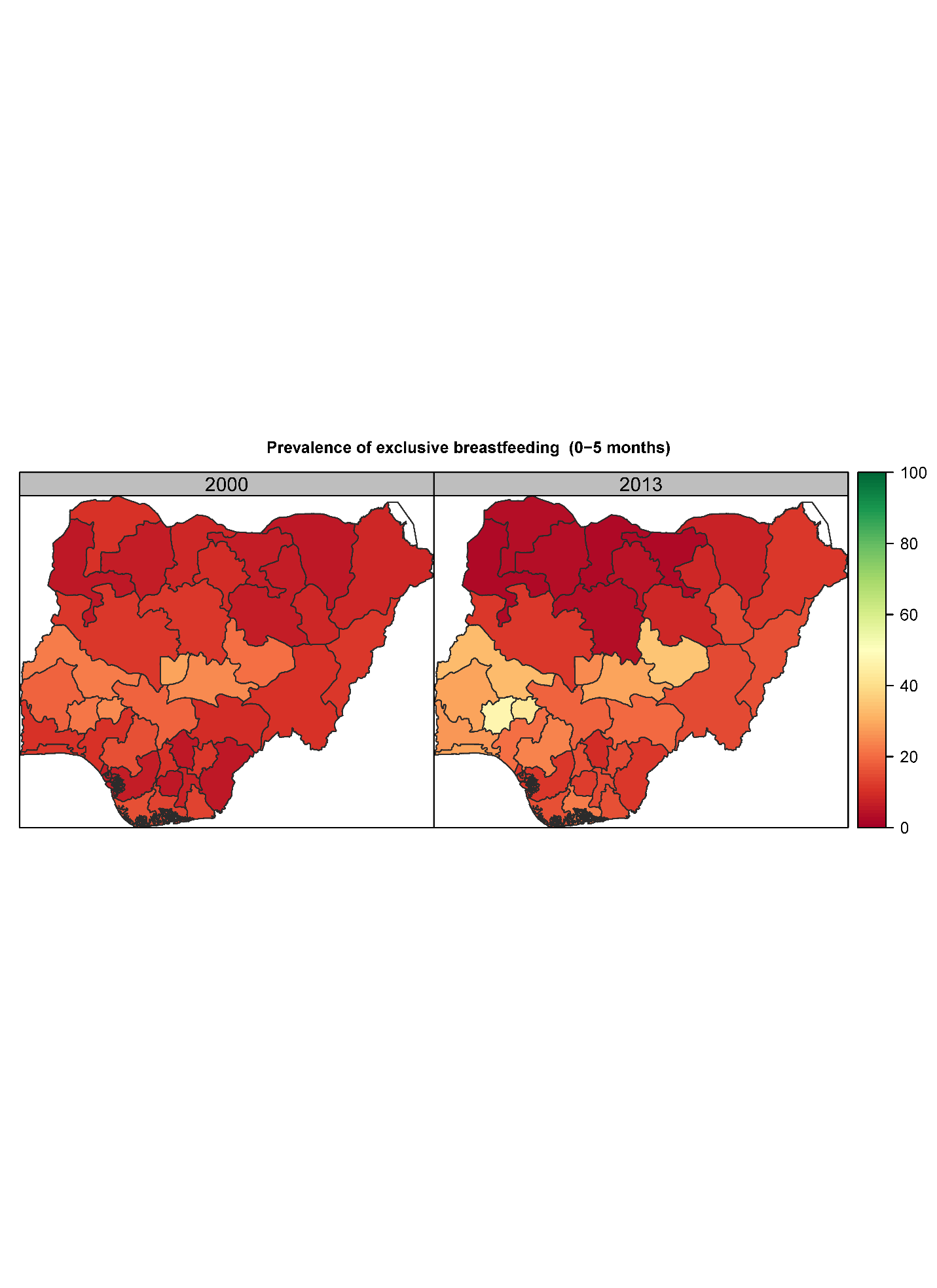
**

**Percentage of women receiving two or more doses of the tetanus toxoid vaccine during pregnancy**

**
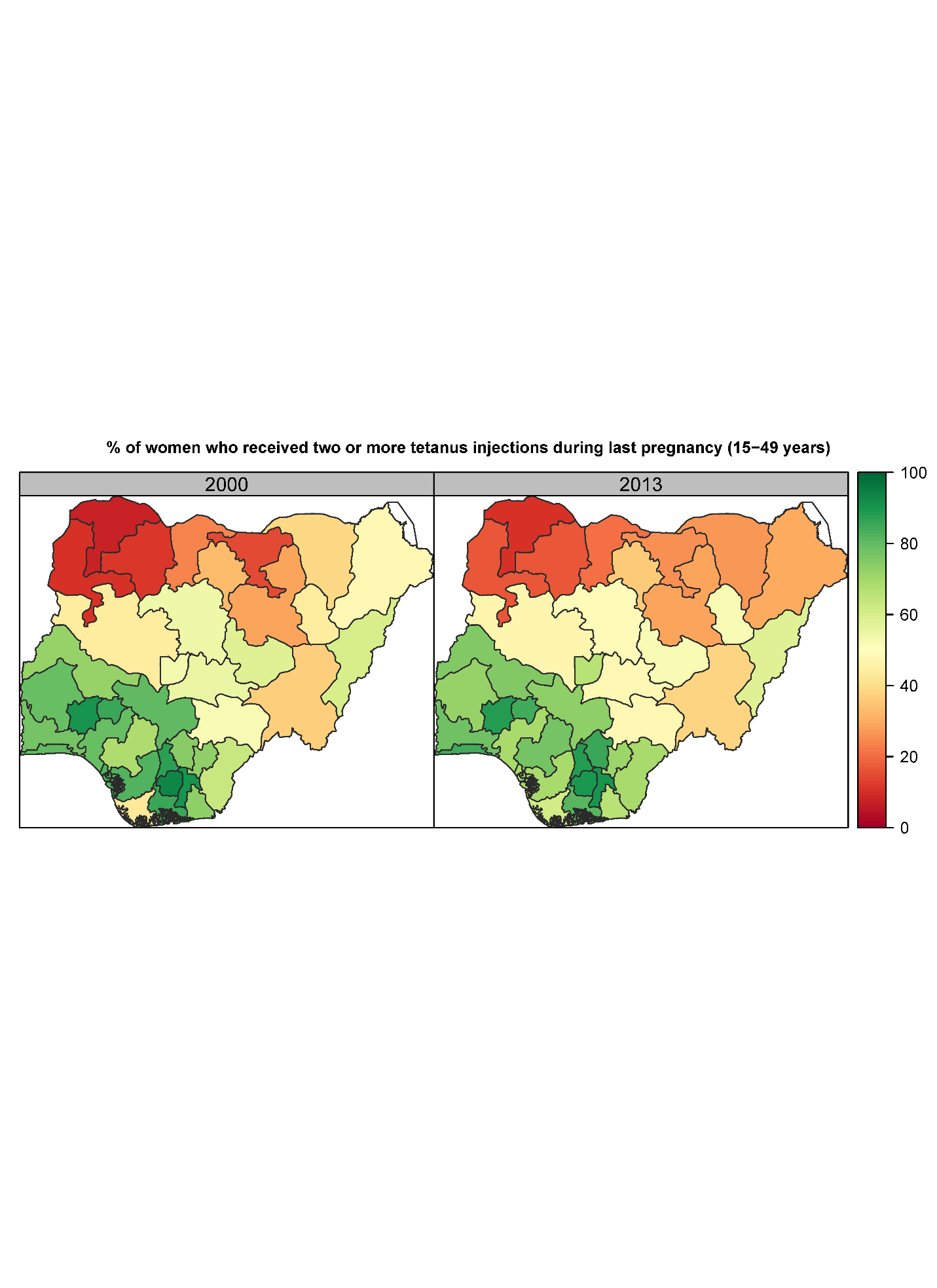
**

**Prevalence of modern contraceptive use (among women aged 15 to 49 years)**

**
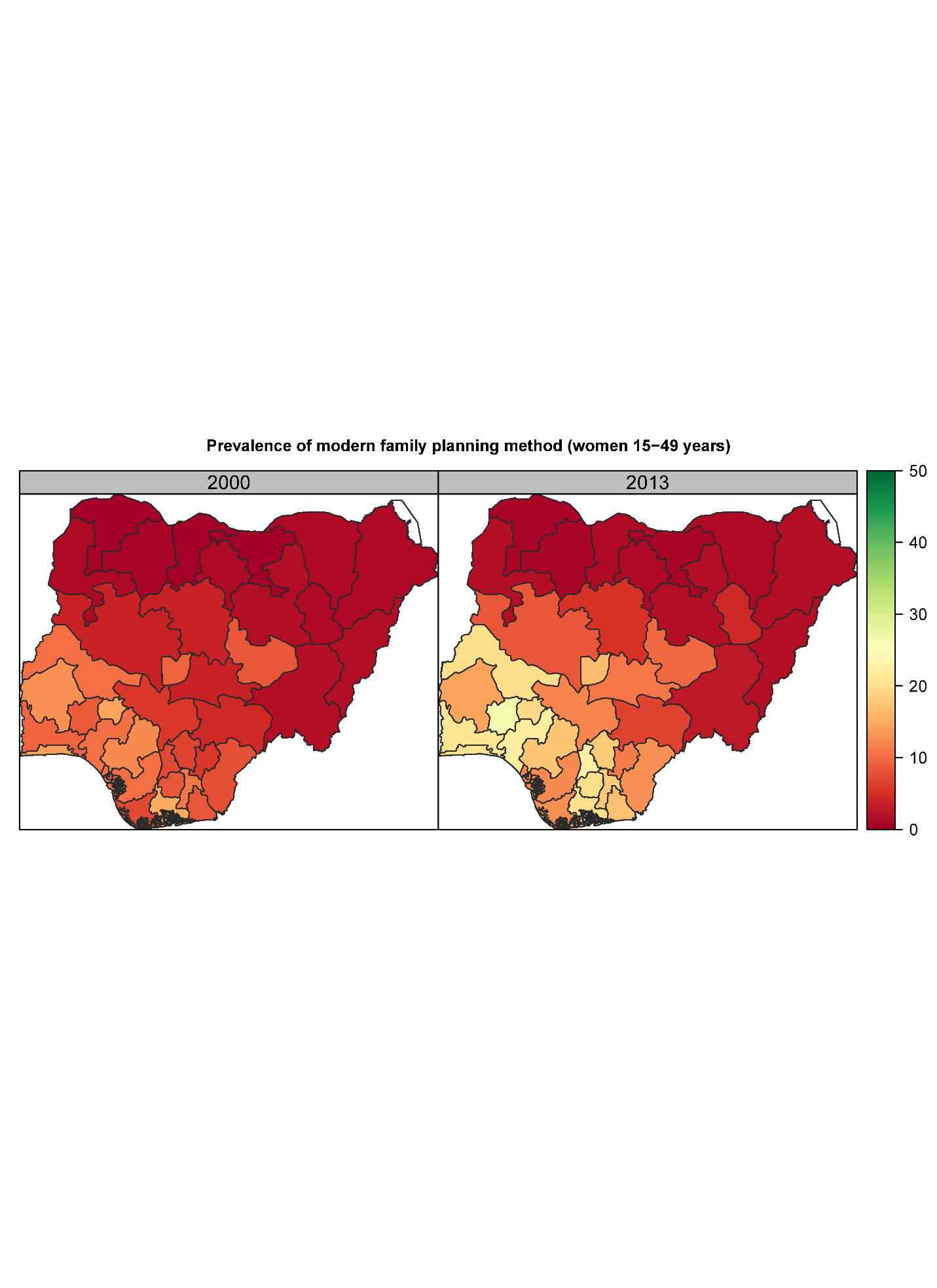
**
